# Factors Associated with Sarcopenia among Elderly Individuals Residing in Community and Nursing Home Settings: A Systematic Review with a Meta-Analysis

**DOI:** 10.3390/nu15204335

**Published:** 2023-10-11

**Authors:** Jia Liu, Yuezhi Zhu, Jen Kit Tan, Azera Hasra Ismail, Roszita Ibrahim, Nor Haty Hassan

**Affiliations:** 1 Department of Nursing, Faculty of Medicine, Universiti Kebangsaan Malaysia, Kuala Lumpur 56000, Malaysia; p129290@siswa.ukm.edu.my (J.L.); azera@ppukm.ukm.edu.my (A.H.I.); 2 Department of Biochemistry, Faculty of Medicine, Universiti Kebangsaan Malaysia, Kuala Lumpur 56000, Malaysia; p118017@siswa.ukm.edu.my (Y.Z.); jenkittan@ukm.edu.my (J.K.T.); 3 Department of Public Health Medicine, Faculty of Medicine, Universiti Kebangsaan Malaysia, Kuala Lumpur 56000, Malaysia; roszita@ppukm.ukm.edu.my

**Keywords:** elderly, sarcopenia, associated factors, community settings, nursing homes, systematic review, meta-analysis

## Abstract

To investigate the factors associated with sarcopenia in elderly individuals residing in nursing homes and community settings, we conducted a systematic search of databases, including MEDLINE, EMBASE, PubMed, Web of Science and Cochrane, up to May 2023. We incorporated a total of 70 studies into our analysis. Our findings revealed that the prevalence of sarcopenia in nursing homes ranged from 25% to 73.7%, while in community settings, it varied from 5.2% to 62.7%. The factors associated with sarcopenia in both nursing homes and community settings included male gender, BMI, malnutrition, and osteoarthritis. In community settings, these factors comprised age, poor nutrition status, small calf circumference, smoking, physical inactivity, cognitive impairment, diabetes, depression and heart disease. Currently, both the European Working Group on Sarcopenia in Older People (EWGSOP) and the Asian Working Group for Sarcopenia (AWGS) standards are widely utilized in nursing homes and community settings, with the EWGSOP standard being more applicable to nursing homes. Identifying factors associated with sarcopenia is of paramount significance, particularly considering that some of them can be modified and managed. Further research is warranted to investigate the impact of preventive measures on these factors in the management of sarcopenia among elderly individuals residing in nursing homes and community settings.

## 1. Introduction

Global aging is a consequence of rapid socioeconomic development and increased life expectancy. In the United States, the population aged 65 and older is projected to rise from 36 million in 2003 to 87 million by 2050 [1]. With the increasing number of elderly individuals, various health challenges arise, including physiological aging, the prevalence of chronic diseases, cognitive decline, malnutrition, reduced physical fitness, and psychosocial issues. Among these geriatric syndromes, sarcopenia stands out as a critical factor that significantly impacts the physical function, health risks, quality of life, and longevity of older adults [2,3].

Sarcopenia refers to the gradual decline in muscle mass and strength that occurs as individuals age. This condition is influenced by various factors, including aging, malnutrition, lack of physical activity, and chronic diseases. However, there is currently no consensus on a universally accepted definition of sarcopenia [4], even though several prominent international organizations, such as the European Working Group on Sarcopenia in Older People (EWGSOP) [5], the Foundation for the National Institutes of Health (FNIH) [6], the International Working Group on Sarcopenia (IWG) and the Asian Working Group on Sarcopenia (AWGS), have published general definitions and guidelines for diagnosing sarcopenia [6,7,8,9] (Table S1).

The onset and progression of sarcopenia are influenced by a multitude of factors. The onset and progression of sarcopenia are influenced by a multitude of factors. Numerous studies have reported that sarcopenia is linked to various factors, including age, physical inactivity, malnutrition, smoking, diabetes, cognitive impairment, heart diseases, and osteoarthritis [10,11,12]. Identifying the factors associated with sarcopenia in both community settings and nursing homes can provide a better understanding of the diverse risk factors across different settings. Furthermore, early detection of these factors in nursing homes and community settings enables timely implementation of preventive measures. These interventions may involve developing personalized activity plans and offering dietary guidance to mitigate further deterioration or the onset of other severe health conditions. These measures ensure that residents in both community and nursing home settings receive the appropriate care and support, ultimately enhancing their quality of life [13,14]. Consequently, we conducted a systematic review and meta-analysis to identify the factors associated with sarcopenia in nursing homes and community settings.

## 2. Materials and Methods

Our study followed the guidelines and principles outlined in the Preferred Reporting Items for Systematic Reviews and Meta-Analyses (PRISMA) statement [15]. Furthermore, the pre-defined review protocol has been registered in the international prospective register of systematic reviews (PROSPERO registration number: CRD42023430442).

### 2.1. Search Strategy

Two researchers independently conducted searches in MEDLINE, EMBASE, PubMed, Web of Science, and the Cochrane Central Register of Controlled Trials from the time of database construction until May 2023. To search these databases, we used the following text words: “elderly”, “aged”, “older adults”, “older people”, “sarcopenia”, “sarcopenic”, “muscle wasting”, “muscle loss”, “muscle weakness”, “risk factors”, “associated factors”, “precipitating factors”, “influence factors”, “contributing factors”, as well as relevant Medical Subject Headings (MeSH) terms.

### 2.2. Inclusion and Exclusion Criteria

We included all studies examining the prevalence and associated factors of sarcopenia in nursing homes and community settings. The age of all participants was required to be 60 years or older. In cases where multiple publications were derived from the same dataset, only the most relevant report was included.

Exclusion criteria based on the guidelines of the four major international organizations (EWGSOP, FNIH, IWG, and AWGS) were applied, including: (1) sarcopenia was defined solely based on biomarkers (e.g., urine creatinine), muscle strength (e.g., grip strength), or anthropometry (e.g., calf circumference or height and weight); (2) absence of explicit reporting on the diagnostic criteria used for sarcopenia; (3) conference abstracts, reviews, editorials, case reports, and letters; (4) non-English publications.

### 2.3. Data Extraction

Data were extracted by one independent reviewer (JL) using the prespecified data extraction forms, and extracted data were verified by a second reviewer (YZZ). Both reviewers independently assessed the inclusion and exclusion criteria for each study. The reasons for excluding each study were recorded in the second step. Discussions were held to resolve any areas of disagreement. The extracted information included study design and methodology, diagnosis criteria of sarcopenia, country, sample demographics (including male and female participants) and mean age, prevalence of sarcopenia, results and conclusions.

### 2.4. Quality Assessment

Two researchers independently assessed the quality level of the included studies. We used the Joanna Briggs Institute Critical Appraisal Checklist for Cross-Sectional Studies [16]. This checklist consists of eight items, each assessed with responses of “not applicable”, “unclear”, “no”, or “yes”. The total count of “yes” responses was recorded for each study. A higher number of “yes” responses indicates higher study quality. Additionally, we employed the Newcastle–Ottawa Scale (NOS) to assess the quality of cohort studies [17]. The NOS consists of three categories with eight items in total, with a maximum score of 9. Studies with a NOS score of 5 or less are considered to have a higher risk of bias, those with scores of 5 to 7 have a moderate risk, and those with scores of 7 or more have a low risk. Any disagreements were arbitrated by a third researcher.

### 2.5. Statistical Analysis

We performed a meta-analysis using Revman 5.4 software, considering *p* < 0.05 as statistically significant. We extracted odds ratios (ORs) and 95% confidence intervals (CIs) for each factor and calculated pooled ORs and 95% CIs. Heterogeneity was assessed using chi-square tests and quantified with the *I*^2^ statistic. If *p* ≤ 0.10 and *I*^2^ ≥ 50%, significant heterogeneity was present, and a random-effects model was employed. Otherwise, a fixed-effects model was used.

## 3. Results

### 3.1. Search Results

**Figure 1:**
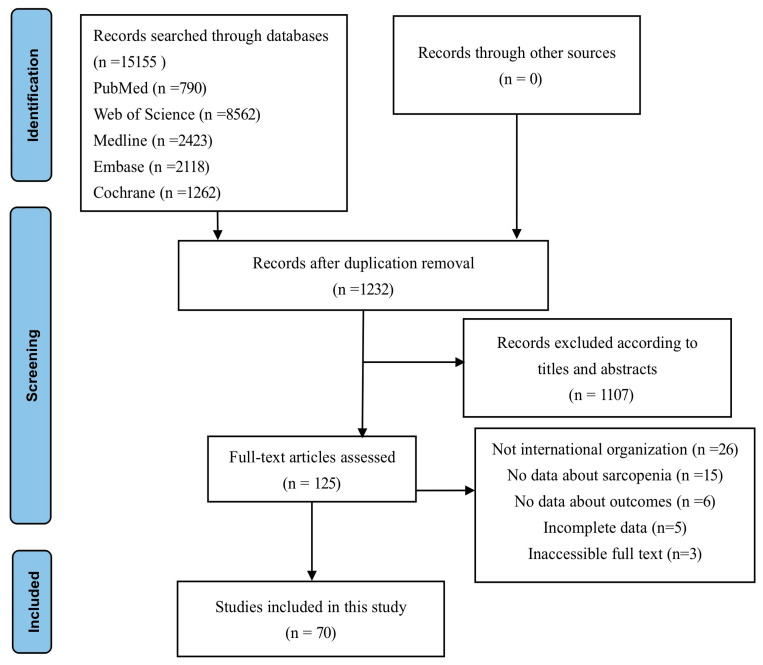
PRISMA diagram for study selection.

In the literature search, we initially identified 15,155 studies, which included 13,923 duplicates. Following a review of titles and abstracts based on the inclusion criteria, we selected 125 studies. After a thorough examination of the full texts, 70 articles were considered suitable for meta-analysis (Figure 1).

### 3.2. Characteristics of Included Studies

In total, 79,328 individuals participated in the studies, consisting of 32,412 males and 41,215 females. Among them, 3249 individuals resided in nursing homes (reported in 11 studies [18,19,20,21,22,23,24,25,26,27,28]), while 76,079 individuals lived in community settings (reported in 58 studies [11,29,30,31,32,33,34,35,36,37,38,39,40,41,42,43,44,45,46,47,48,49,50,51,52,53,54,55,56,57,58,59,60,61,62,63,64,65,66,67,68,69,70,71,72,73,74,75,76,77,78,79,80,81,82,83,84,85]). Notably, five studies [50,51,60,65,72] did not differentiate between males and females, while two studies [45,63] specifically focused on females.

Out of the total studies, 52 [11,18,20,21,22,24,25,26,27,28,29,32,33,34,35,36,38,39,41,42,45,46,47,48,49,50,51,53,56,60,61,63,64,66,67,68,69,70,71,72,73,74,75,76,77,78,79,80,81,83,84,85] followed a cross-sectional design, while the remaining 17 [19,23,28,30,31,37,40,43,44,52,54,55,58,59,62,65,82] were cohort studies. Among these studies, 48 [11,23,25,26,27,28,29,31,32,33,34,35,36,37,38,39,40,46,47,48,49,50,51,52,53,54,55,56,57,59,60,61,62,63,65,66,70,71,74,75,77,78,80,81,82,83,84,85] were conducted in Asian countries, and 21 were from non-Asian countries (10 from Europe [18,19,20,21,22,43,44,58,76,79], 9 from the Americas [30,42,45,64,67,68,69,72,73], 1 from Australia [24] and 1 from Oceania [41]). 

Regarding the criteria used, the EWGSOP criteria (n = 38) and AWGS criteria (n = 32) were the most commonly employed standards. Specifically, 8 nursing home studies and 29 community studies used the EWGSOP criteria, while 1 nursing home study and 30 community studies used the AWGS criteria. Two studies utilized the updated EWGSOP2 criteria, and one study [27] employed a combination of four international diagnostic criteria: EWGSOP, EWGSOP2, AWGS, and IWGS. The characteristics of the 70 included studies are presented in Table S2.

### 3.3. Quality Assessment of the Included studies

The Joanna Briggs Institute Critical Appraisal Checklist was used to evaluate the quality of the 53 cross-sectional studies. Of these, 6 studies met six checklist items, 18 studies met seven items, and the remaining 29 studies met all eight items. For the 18 cohort studies, the Newcastle–Ottawa Scale was used to assess quality. One study scored 8 and the remaining 17 studies scored 9, indicating high quality. The quality assessment results for the cross-sectional and cohort studies are presented in Figures S1 and S2 and Figures S3 and S4, respectively.

### 3.4. Prevalence and Associated Factors of Sarcopenia of Older Adults

In the included studies of older individuals in nursing homes and communities, the prevalence of sarcopenia in nursing homes ranged from 25% to 73.7%, while in the community, it ranged from 5.2% to 62.7%. Associated factors were categorized into social demographic factors (age and gender), physiological factors (calf circumference, BMI, malnutrition, and nutrition status), behavioral factors (smoker and physical inactivity), and disease factors (cognitive impairment, diabetes, depression, heart diseases, and osteoarthritis) (Table 1). 

#### 3.4.1. Associated Factors of Sarcopenia in Nursing Homes

##### Socio-Demographic Factors

The meta-analysis findings indicated that male gender (two studies, OR = 6.35, 95% CI: 3.62–11.14, Table 1, Figure S5) was identified as an associated factor for sarcopenia in nursing homes.

##### Physiological Factors

The meta-analysis findings indicated that sarcopenia and BMI were negatively associated (four studies, OR = 0.76, 95% CI: 0.67–0.86, Table 1, Figure S6), while sarcopenia and malnutrition were positively associated (four studies, OR = 3.20, 95% CI: 1.65–6.21, Table 1, Figure S7) among elderly individuals residing in nursing homes.

##### Disease Factors

The meta-analysis findings indicated a positive association between sarcopenia and osteoarthritis (two studies, OR = 3.17, 95% CI: 0.83–12.11, Table 1, Figure S8) in nursing homes.

#### 3.4.2. Associated Factors of Sarcopenia in Community Settings

##### Socio-Demographic Factors

In the community settings, there was a positive association between sarcopenia and age (34 studies, OR = 1.15, 95% CI: 1.10–1.19, Table 1, Figure S9). Male gender was positively associated with sarcopenia (seven studies, OR = 2.77, 95% CI: 1.63–4.69, Table 1, Figure S10), while female gender was negatively associated with sarcopenia (three studies, OR = 0.49, CI: 0.09–2.55, Table 1, Figure S11) in the community settings.

##### Physiological Factors

There was a negative association between sarcopenia and l BMI (23 studies, OR = 0.83, 95% CI: 0.68–1.02, Table 1, Figure S12) in the community settings. Sarcopenia and malnutrition were positively associated (nine studies, OR = 2.62, 95% CI: 1.47–4.67, Table 1, Figure S13). Sarcopenia and poor nutrition status were negatively associated (three studies, OR = 0.81, CI: 0.74–0.99, Table 1, Figure S14). Sarcopenia and calf circumference were negatively associated (five studies, OR = 0.56, 95% CI: 0.38–0.83, Table 1, Figure S15) in community settings.

##### Behavioral Factors

The meta-analysis findings indicated that sarcopenia is positively associated with smoking (4 studies, OR = 2.08, 95% CI: 1.53–2.84, Table 1, Figure S16) and physical inactivity (10 studies, OR = 2.03, 95% CI: 1.60–2.56, Table 1, Figure S17) in community settings.

##### Disease Factors

In community settings, there was a positive association between sarcopenia and cognitive impairment (9 studies, OR = 2.10, 95% CI: 1.54–2.87, Table 1, Figure S18), diabetes (16 studies, OR = 2.33, 95% CI: 1.49–3.62, Table 1, Figure S19), depression (6 studies, OR = 1.91, 95% CI: 1.62–2.25, Table 1, Figure S19), heart disease (3 studies, OR = 1.81, 95% CI: 0.85–3.86, Table 1, Figure S21), and osteoarthritis (6 studies, OR = 1.59, 95% CI: 1.30–1.93, Table 1, Figure S22).

#### 3.4.3. Associated Factors of Sarcopenia in Both Nursing Homes and Community Settings 

##### Socio-Demographic Factors

When combing data from both community settings and nursing homes, the meta-analysis findings indicated that male gender (nine studies, OR = 3.39, 95% CI: 2.05–5.62, Table 1, Figure S23) was positively associated with sarcopenia.

##### Physiological Factors

The meta-analysis findings indicated that sarcopenia was negatively associated with BMI (27 studies, OR = 0.80, 95% CI: 0.67–0.96, Table 1, Figure S24), positively associated with malnutrition (13 studies, OR = 2.82, 95% CI: 1.80–4.40, Table 1, Figure S25), and negatively associated with calf circumference (6 studies, OR = 0.63, 95% CI: 0.43–0.94, Table 1, Figure S26) in both community settings and nursing homes.

##### Behavioral Factors

There was a positive association between sarcopenia and smoking (five studies, OR = 2.06, 95% CI: 1.56–2.73, Table 1, Figure S27) in both community settings and nursing homes.

##### Disease Factors

Sarcopenia and osteoarthritis were positively associated (eight studies, OR = 1.69, 95% CI: 1.38–2.08, Table 1, Figure S28) in both community settings and nursing homes.

## 4. Discussion

According to our study, the prevalence of sarcopenia in nursing homes ranged from 25% to 73.7%, whereas in community settings, it ranged from 5.2% to 62.7%. This finding was corroborated by a previous study [23]. This difference may be attributed to the generally poorer health conditions of elderly individuals residing in nursing homes compared to those living in the community. Residents in nursing homes often contend with a combination of illnesses that can lead to muscular atrophy, increasing the risk of sarcopenia. Furthermore, due to mobility limitations, frailty, and stricter regulations in nursing homes compared to the general community, residents in these facilities may have limited opportunities for physical activity. Infrequent exercise can result in muscle mass loss and an elevated risk of muscular dystrophy [88,89]. Additionally, we hypothesize that sarcopenia in nursing home residents may manifest at a later stage compared to community settings.

Additionally, our study reveals that the EWGSOP criteria, which are better suited for use with nursing home residents, are the most commonly employed criteria, followed by the AWGS criteria. In contrast, the IWGS, EWGSOP2, and FNIH criteria are less frequently utilized (Table S1). In our analysis, only one nursing home study applied all of the EWGSOP, IWGS, AWGS, and FNIH criteria for diagnosis [6,7]. Applying multiple diagnostic criteria to the same patient group will result in varying prevalence rates of sarcopenia [90]. Regarding these criteria, the EWGSOP and AWGS criteria have previously been proposed and validated to account for variations in the characteristics of older populations from diverse geographic and cultural backgrounds, making them more relevant to Europe and Asia. In contrast, the IWGS and FNIH working groups have primarily focused on Europe and the United States [7].

### 4.1. Sociodemographic Factors Associated with Sarcopenia in Nursing Homes and Community Settings

According to our findings, male gender is a sociodemographic factor positively associated with the occurrence of sarcopenia among residents of nursing homes and community settings. However, the literature remains divided on whether gender has a causal relationship with sarcopenia [91]. Some studies have shown that men are more prone to developing sarcopenia in nursing homes and community settings [26,34,35,49,56,57,66]. Gallagher et al. indicated that men experience a rate of muscle loss twice as fast as women [92]. This could be attributed to the fact that men over the age of 30 undergo an annual decrease of 1–2% in both total testosterone levels and bioavailable testosterone levels, leading to a reduction in skeletal muscle mass and quantity [93]. Estrogen also plays a crucial role in preserving skeletal muscles contractility and preventing muscular injury [88]. Despite the decline in estrogen levels with age among women, they consistently maintain significantly higher levels of the hormone throughout the lifespan compared to testosterone levels in men. 

In contrast, some studies [39,47,61,73] have suggested that females are at higher risk of developing sarcopenia. This could be due to male participants in the studies being more likely to engage in physical labor, resulting in stronger grips and faster walking speeds compared to female participants. However, there are studies that have reported no difference in the prevalence of sarcopenia between men and women [58,92,94]. Further research is needed to confirm the causal relationship between gender and sarcopenia. 

Our findings have indicated that age has a positive association with sarcopenia in community settings. These findings are in line with previous studies [7,31]. However, our findings do not show age is a significant factor associated with sarcopenia in nursing homes. It is possible that age is no longer the primary factor driving the development of sarcopenia in nursing home residents because age-related factors, such as disease burden, nutritional status, medication use, and other comorbidities, may have a greater impact than age itself. Moreover, nursing homes prioritize the recovery and rehabilitation of their residents, often providing pharmacological therapy, dietary supplementation, and rehabilitation training to address sarcopenia. These interventions could mitigate the association between age and sarcopenia and reduce the impact of aging on the development of sarcopenia among elderly individuals in nursing homes.

### 4.2. Physiological Factors Related to Sarcopenia in Nursing Homes and Community Settings

Our findings suggest a negative association between sarcopenia and high BMI, alongside a positive association with malnutrition in both nursing homes and community settings. A strong correlation exists between the occurrence of sarcopenia and the skeletal muscle mass index (SMI), which has a positive relationship with BMI [43]. A higher BMI, leading to increased SMI, suggests better nutritional intake [35]. Zhang at al. demonstrated that a higher BMI resulted in reduced sarcopenia and muscle mass loss during a four-year follow-up [95]. In both community settings and nursing homes, the majority of the population comprises elderly individuals, who may face challenges in obtaining adequate nutrition for various reasons. These challenges include difficulty in chewing, swallowing, or having missing teeth, which can make eating a challenge. Additionally, dysbiosis of the gastrointestinal flora can reduce amino acid biosynthesis and the secretion of short-chain fatty acids, impacting the activity of mitochondria in skeletal muscle cells, and consequently, leading to skeletal muscle remodeling or atrophy [96]. Nevertheless, the excessive accumulation of visceral fat can contribute to the development of the sarcopenic obesity phenotype [61]. This phenotype is characterized by a combination of obesity and a loss of muscle mass, with an increase in adipose tissue and a relative deficit in skeletal muscle. Obesity can also negatively impact bodily function, metabolism, and overall health, increasing the risk of conditions such as diabetes and cardiovascular disease [97]. 

Our meta-analysis shows that good nutritional status is negatively associated with sarcopenia in community settings. There are three community-based studies [98,99,100] have investigated the association between nutrition status and sarcopenia in community settings. These studies emphasized the role of vitamin D and leucine-rich whey protein in promoting myogenic protein synthesis among elderly individuals who were not protein-malnourished but performed limited physical activity. Vitamin D plays a crucial role in the crosstalk between skeletal muscle and bone by stimulating the production of myogenic factors derived from both bone and skeletal muscle. Vitamin D deficiency can impair skeletal muscle-bone crosstalk, negatively affecting muscle function, repair, chondrocyte maturation, bone mineralization, and increasing bone resorption, through alterations in calcium and phosphate metabolism [96].

Additionally, our findings show that small calf circumference can predict sarcopenia exclusively in community settings, rather than in nursing homes. Although the same diagnostic criteria for sarcopenia were applied, differences in the techniques used to measure muscle mass and function may account for this discrepancy between community settings and nursing homes [27]. For instance, Senior et.al. used Bioelectrical Impedance Analysis (BIA) [23], while Saka et al. [24] assessed muscle mass using calf circumference. Moreover, it is worth noting that calf circumference among elderly individuals can exhibit some degree of variation across different countries. For example, variations exist between the 35 cm criterion established Tasar et al. in a Turkish population [26] and the 31 cm threshold recommended by the World Health Organization for elderly individuals.

### 4.3. Behavioral Factors Related to Sarcopenia in Nursing Homes and Community Settings

According to the findings of our study, smoking and physical inactivity have a positive association with sarcopenia in community settings only. Smokers, compared to non-smokers, have lower cross-sectional areas of type I muscle fibers, worsened oxidative fiber atrophy, increased glycolytic capacity, and rapid protein deterioration, leading to a reduction in muscular performance [101,102]. Smoking also impairs mitochondrial oxygen transport, prevents the synthesis of adenosine triphosphate (ATP), alters the function of skeletal muscle contraction, and increases the expression of genes associated with muscle injury [103,104,105]. Additionally, a study [102] discovered that smokers showed significant gains in skeletal muscle fatigue resistance and a declining trend in inflammatory responses two weeks after quitting smoking. 

Prolonged periods of physical inactivity can reduce insulin sensitivity, increase muscle and fat infiltration, and deteriorate muscle function. In one study, elderly individuals who engaged in resistance and balance training twice a week showed improvements in grip strength and gait speed, along with a decrease in body fat percentage. Notably, the incidence of sarcopenia did not increase among those who participated in this training regimen, while in the control group that maintained their usual lifestyle, the incidence of sarcopenia rose from 42.9% to 52.4% [105]. This serves as further evidence that regular exercise can increase muscle strength, balance, preserve joint flexibility, and promote neural protection and regeneration, all of which can lower the risk of developing sarcopenia [59]. However, our findings do not show physical inactivity is associated with sarcopenia in nursing homes. This may be explained by the increased propensity for senior residents of nursing homes to go through rehabilitation training and follow a healthy diet.

### 4.4. Disease-Related Factors of Sarcopenia in Nursing Homes and Community Settings

According to our findings, osteoarthritis is positively associated with sarcopenia in elderly individuals in nursing homes and community settings. Other significant associated factors for sarcopenia among the elderly individuals in the community include cognitive impairment, diabetes, heart disease, and depression. Patients with osteoarthritis may have joint discomfort and stiffness that limit their ability to move, ultimately leading to muscle atrophy and a loss of function [106]. Pathological changes in muscle fiber activity are present in elderly people with cognitive impairment [107], and these changes result in muscular atrophy and diminished function [28,108]. Diabetes patients may experience oxidative stress, chronic inflammation, and insulin resistance, all of which impair the synthesis of muscle protein and fail to adequately supply energy to muscle cells [109]. In the elderly individuals with heart disease, the release of inflammatory mediators and activation of the immune system due to cardiac tissue damage frequently result in chronic inflammation, which increases the levels of inflammatory substances in muscle tissue and, ultimately, reduces muscle protein synthesis and impairs muscle function [109]. Additionally, there is a direct association between sarcopenia and sadness [110]. Depression can lead to diminished physical activity, decreased mobility, and reduced nutritional intake, which can result in the deterioration of muscle function and strength.

### 4.5. Recommendations

Based on our findings, it is recommended that healthcare providers propose a comprehensive set of recommendations to address sarcopenia in elderly individuals, whether they reside in community or nursing home settings. Firstly, healthcare professionals should implement preventive measures, irrespective of the patient’s gender. Secondly, they should ensure uniform training for health information collectors to standardize measurements of health parameters, such as calf circumference, to minimize variations in data analysis. Thirdly, they should recommend nutrient supplementation, including vitamin D and protein, for elderly individuals to enhance muscle mass. Fourth, they should encourage smoking cessation among elderly individuals, provide information about smoking’s adverse effects on muscle health, and consider alternative therapies like nicotine gum replacement therapy to support smokers in quitting. Fifth, providers should develop personalized exercise plans for seniors, and offer health lectures and educational programs focused on muscle health to raise awareness about the benefits of exercise and a balanced diet. Additionally, they should encourage seniors to engage in various social, cultural, and recreational activities. Lastly, the importance of prioritizing chronic disease management should be emphasized among elderly individuals. Regular check-ups and monitoring are vital for early detection and intervention in worsening chronic conditions, thereby alleviating symptoms and improving treatment outcomes.

## 5. Limitation

Our study has limitations. There is a limited number of studies on sarcopenia in nursing homes, and most of the studies conducted in both nursing homes and community settings so far have employed cross-sectional designs, making it impossible to establish causal relationships between associated factors and sarcopenia. To address this limitation, conducting a longitudinal cohort study would be valuable in exploring the differential factors contributing to sarcopenia in nursing homes and community settings.

## 6. Conclusions

Many factors are associated with sarcopenia, and some of them are modifiable. Our meta-analysis indicates that male gender, BMI, malnutrition and osteoarthritis are positively associated with sarcopenia in both nursing homes and community settings. Additionally, age, poor nutritional status, reduced calf circumference, smoking, physical inactivity, cognitive impairment, diabetes, depression and heart diseases are positively associated with sarcopenia in community settings. Interestingly, our findings show that female gender is negatively associated with sarcopenia in community settings. There is an urgent need to manage sarcopenia due to its high prevalence in nursing homes and community settings. Identifying the factors associated with sarcopenia enables the planning of personalized preventive measures for early interventions. Further research is required to validate the causal relationship between these factors and sarcopenia.

## Figures and Tables

**Table 1:** Pooled odds ratio and 95% confidence intervals for factors associated with sarcopenia in nursing homes and community settings.

Associated Factors		Setting	Number of Studies	Heterogeneity		OR (95% CI)
				*I* ^2^	*p*	
Sociodemographic factors	Gender (male)	NH	2	32.0	0.230	6.35 (3.62–11.14)
Physiological factors	BMI	NH	4	0.0	0.530	0.76 (0.67–0.86)
	Malnutrition *	NH	4	51.0	0.110	3.20 (1.65–6.21)
Disease factors	Osteoarthritis	NH	2	75.0	0.05	3.17 (0.83–12.11)
Sociodemographic factors	Age	Community	34	90.0	<0.001	1.15 (1.10–1.19)
	Gender (male)	Community	7	77.0	<0.001	2.77 (1.63–4.69)
	Gender (female)	Community	3	94.0	<0.001	0.49 (0.09–2.55)
Physiological factors	BMI	Community	23	96.0	<0.001	0.83 (0.68–1.02)
	Malnutrition *	Community	9	90.0	<0.001	2.62 (1.47–4.67)
	Poor nutrition status **	Community	3	0.0	0.530	0.81 (0.74–0.99)
	Calf circumference	Community	5	95.0	<0.001	0.56 (0.38–0.83)
Behavioral factor	Smoking	Community	4	0.0	0.950	2.08 (1.53–2.84)
	Physical inactivity	Community	10	60.0	0.007	2.03 (1.60–2.56)
Disease factor	Cognitive impairment	Community	9	55.0	0.020	2.10 (1.54–2.87)
	Diabetes	Community	6	81.0	<0.001	2.33 (1.49–3.62)
	Depression	Community	6	0.0	0.750	1.91 (1.62–2.25)
	Heart diseases	Community	3	76.0	0.020	1.81 (0.85–3.86)
	Osteoarthritis	Community	6	67.0	0.009	1.59 (1.30–1.93)
Sociodemographic factors	Gender (male)	NH and Community	9	79.0	<0.001	3.39 (2.05–5.62)
Physiological factors	BMI	NH and Community	27	95.0	<0.001	0.80 (0.67–0.96)
	Malnutrition *	NH and Community	13	88.0	<0.001	2.82 (1.80–4.40)
	Calf circumference	NH and Community	6	94.0	<0.001	0.63 (0.43–0.94)
Behavioral factor	Smoking	NH and Community	5	0.0	0.980	2.06 (1.56–2.73)
Disease factor	Osteoarthritis	NH and Community	8	69.0	0.002	1.69 (1.38–2.08)

BMI: Body Mass Index; CI: confidence interval; *I*^2^: I-squared statistic; NH: nursing home; OR: odds ratio * Malnutrition: a condition where the body cannot meet its health requirements due to insufficient or imbalanced dietary intake. Malnutrition is often associated with poor nutritional status [86]; ** Nutrition status: a set of indicators used to assess the nutritional health of individuals or populations, including body weight, height, BMI, nutrient levels, dietary intake, and clinical evaluation [87].

## Data Availability

Not applicable.

## Supplementary Materials

The following supporting information can be downloaded at: https://www.mdpi.com/article/10.3390/nu15204335/s1, Figure S1: Risk of bias graph of the included cross-sectional studies; Figure S2: Risk of bias summary of the included cross-sectional studies; Figure S3: Risk of bias graph of the included cohort studies; Figure S4: Risk of bias summary of the included cohort studies; Figure S5: Forest plot of the association between male and sarcopenia in NH; Figure S6: Forest plot of the association between BMI and sarcopenia in NH; Figure S7: Forest plot of the association between malnutrition and sarcopenia in NH; Figure S8: Forest plot of the association between osteoarthritis and sarcopenia in NH; Figure S9: Forest plot of the association between age and sarcopenia in community settings; Figure S10: Forest plot of the association between male and sarcopenia in community settings; Figure S11: Forest plot of the association between female and sarcopenia in community settings; Figure S12: Forest plot of the association between BMI and sarcopenia in community settings; Figure S13: Forest plot of the association between malnutrition and sarcopenia in community settings; Figure S14: Forest plot of the association between nutrition status and sarcopenia in community settings; Figure S15: Forest plot of the association between calf circumference and sarcopenia in community settings; Figure S16: Forest plot of the association between smoking and sarcopenia in community settings; Figure S17: Forest plot of the association between physical inactivity and sarcopenia in community-settings; Figure S18: Forest plot of the association between cognitive impairment and sarcopenia in community settings; Figure S19: Forest plot of the association between diabetes and sarcopenia in community settings; Figure S20: Forest plot of the association between depression and sarcopenia in community settings; Figure S21: Forest plot of the association between heart diseases and sarcopenia in community settings; Figure S22: Forest plot of the association between osteoarthritis and sarcopenia in community settings; Figure S23: Forest plot of the association between male and sarcopenia in nursing home and community settings; Figure S24: Forest plot of the association between BMI and sarcopenia in nursing home and community settings; Figure S25: Forest plot of the association between malnutrition and sarcopenia in nursing home and community settings; Figure S26: Forest plot of the association between calf circumference and sarcopenia in nursing home and community settings; Figure S27: Forest plot of the association between smoking and sarcopenia in nursing home and community settings; Figure S28: Forest plot of the association between osteoarthritis and sarcopenia in nursing home and community settings.; Table S1: Definition and cut-off points of sarcopenia; Table S2: Characteristics of studies included in this meta-analysis.
