# Supplementary material for: Factors Associated with Sarcopenia among Elderly Individuals Residing in Community and Nursing Home Settings: A Systematic Review with a Meta-Analysis
Source: Nutrients. 2023 Oct 11;15(20):4335. doi: 10.3390/nu15204335 (PMC10610239; doi:10.3390/nu15204335)

Table S1 Definition and cut-off points of sarcopenia.

Classification	Definition	Muscle mass	Muscle strength		No. of studies (n)
		ASM/height2 (kg/m2)	Grip strength (kg)	Gait speed (m/s)	
EWGSOP	● Low muscle mass<br>● Low grip strength or slow gait speed	● Men < 7.23-7.26<br>● Women < 5.45-5.67	● Men < 30<br>● Women < 20	< 0.8	39
AWGS	● Low muscle mass<br>● Low grip strength or slow gait speed	● Men < 7<br>● Women < 5.4	● Men < 26<br>● Women < 18	< 0.8	32
EWGSOP2	● Low muscle mass<br>● Low grip strength	● Men < 7.0<br>● Women < 5.5	● Men < 27<br>● Women < 16	≤0.8	2
IWG	● Low muscle mass<br>● Slow gait speed	● Men < 7.23<br>● Women < 5.67	-	≤1.0	0
FNIH	● Low muscle mass<br>● Low grip strength	● Men < 19.75<br>● Women < 15.02	● Men < 26<br>● Women < 16	< 0.8	0

Table S2 Characteristics of studies included in this meta-analysis.

First author(year)	Setting	Region	Study design	Total N	Male N	Female N	Mean age	Age	Criteria	Prevalence (N/%)	Results
RODRIGUEZ-REJON 2018	NH	Spain	cross-sectional study	249	62	187	84.9± 6.7	⩾60	EWGSOP	157（63%）	Sarcopenia prevalence: EWGSOP, Algorithm A, and Algorithm B yielded consistent rates of 63.0%, 62.9%, and 63.2%.
Buckinx 2017	NH	Belgium	cohort study	662	178	484	83.2±8.99	⩾60	EWGSOP	252(38.1%)	A 38.1% prevalence of sarcopenia and nearly 50% of frail individuals being diagnosed as sarcopenic.
Anna 2022	NH	Spain	cross-sectional study	104	16	88	84.6±7.8	⩾60	EWGSOP2	26(25%)	High sarcopenia prevalence (6.7%-81.7%) per EWGSOP2 criteria; linked to malnutrition, incontinence, sedentary behavior; obesity protective.
Francesco 2012	NH	Italy	cross-sectional study	122	31	91	84.1±6.9	⩾60	EWGSOP	40(32.8%)	Sarcopenia: Prevalent in 32.8%, higher in males, cerebrovascular disease, osteoarthritis, and lower BMI; physical activity lowers risk.
Lardies-Sanchez 2017	NH	Spain	cross-sectional study	339	121	218	84.9±7.6	⩾60	EWGSOP	129(38.1%)	Sarcopenia: Prevalent in 38.1% of nursing home residents, associated with malnutrition, low BMI, and age over 80, especially in women.
Saka 2016	NH	Turkey	Cohort study	401	199	203	74.9±6.8	⩾60	EWGSOP	294(73.3%)	Malnutrition, sarcopenia common in elderly nursing home residents, associated with increased independent mortality risk.
Senior 2015	NH	Australia	cross-sectional study	102	-	-	84.5±8.2	⩾60	EWGSOP	41(40.2%)	High sarcopenia prevalence (40.2%) in aged care residents; low BMI predicts sarcopenia.
Sun 2023	NH	China	cross-sectional study	583	204	333	86.0±6.6	⩾60	AWGS	384(65.9%)	High malnutrition risk (47.9%) among elderly in senior homes. Dysphagia, sarcopenia, dementia linked; exercise reduces risk.
Tasar 2015	NH	Turkey	cross-sectional study	211	87	124	77.3±7.20	⩾60	EWGSOP	71(33.6%)	Sarcopenia prevalence: 33.6% in Turkish nursing home residents, higher in men, young-old, and those with specific health conditions.
Zeng 2018	NH	China	cross-sectional study	277	83	194	81.6±3.3	⩾60	EWGSOP, AWGS、IWGS 和 FNIH	32.5%,34.3%,38.3% and 31.4%	Sarcopenia is prevalent in Chinese nursing home residents (32.5% to 38.3%). Malnutrition and calf circumference relate to sarcopenia.
Yalcin 2016	NH	Turkey	cross-sectional study	141	62	79	79.1±7.99	⩾60	EWGSOP	41(29%)	Sarcopenia prevalence in Turkish nursing home residents is 29%. Sarcopenia independently increases all-cause mortality, influenced by nutritional status.
Liu 2020	Community	China	cross-sectional study	4500	1627	2873	62.4±8.3	⩾60	EWGSOP	869(19.31%)	Sarcopenia prevalence varied among ethnic groups in west China, with Qiang having a lower prevalence compared to Han. Further research needed.
Dasilvaalexandre 2014	Community	Brazil	cohort study	1149	473	712	71.9±0.8	⩾60	EWGSOP	266(23.15%)	High sarcopenia prevalence (14.4% in men, 16.1% in women) in São Paulo's older population, associated with various factors. No gender differences observed.
Zhang 2020	Community	China	cohort study	474	238	236	68.1±6.19	⩾60	AWGS	73(15.4%)	Aging, gender, stroke, and BMI influence muscle loss in elderly Chinese sarcopenia patients over 4 years.
Yao 2022	Community	China	cross-sectional study	1082	466	616	76.6±7.11	⩾60	AWGS	520(48.06%)	Prevalent possible sarcopenia in older Chinese adults with gender-specific risk factors, suggesting targeted preventive measures.

Xu 2020	Community	China	cross-sectional study	582	247	335	86.4±3.5	⩾80	AWGS	155（26.6%）	Sarcopenia is prevalent among Chinese oldest old, linked to age, lower BMI, nutritional status, and associated with disability and poor physical function.
Manal 2020	Community	Palestine	cross-sectional study	145	72	78	69.5±5.7	⩾60	EWGSOP	39（26.9%）	Sarcopenia is prevalent in Hebron, Palestine, with a higher rate in men, and it's associated with BMI, cognitive and functional impairment, and depressive symptoms. Further research is needed.
Chang 2021	Community	China	cross-sectional study	170	42	128	81.6± 6.8	⩾65	AWGS	88(51.76%)	Sarcopenia prevalence is high among daycare center attendees in Taiwan, with factors like calf circumference, nutritional status, dementia, BMI, and gait speed associated with it. Early interventions are crucial.
Chen X 2021	Community	China	cross-sectional study	938	462	276	70.1±6.61	⩾60	AWGS	172(18.34%)	In Chengdu, China, sarcopenia is prevalent, with higher rates in males. Age and cognitive impairment are significant factors in females, while age, BMI, smoking, and COPD play roles in males, especially in severe sarcopenia.
Chen Z 2021	Community	China	Cohort study	4866	2448	2418	67.7±6.4	⩾60	AWGS	2238(46%)	A high prevalence (46.0%) and incidence (11.9/100 person-years) of possible sarcopenia in older adults, emphasizing the need for early detection and intervention.
Cheng 2021	Community	China	cross-sectional study	390	164	226	78.1±7.4	⩾60	AWGS	225(57.7%)	A significant proportion of older adults living alone in China are at risk of sarcopenia, emphasizing the need for early intervention and lifestyle improvements.
Chew 2022	Community	Singapore	cross-sectional study	694	265	429	73.4±0.3	⩾65	AWGS	137(19.74%)	Sarcopenia prevalence is high in older adults at risk of malnutrition, emphasizing the need for early interventions.
Choe 2022	Community	Korea	Cohort study	1636	746	890	76.6±3.75	⩾70	AWGS	205(12.53%)	High sarcopenia rates in malnourished older adults underscore the importance of early intervention.
Darroch 2022	Community	New Zealand	cross-sectional study	91	33	58	86.0±8.3	⩾65	EWGSOP	37(41%)	Sarcopenia prevalence was 41% in New Zealand RAC residents, with malnutrition risk and lower BMI as significant risk factors.
de Souza 2022	Community	Brazil	cross-sectional study	306	130	176	69.87±7.06	⩾60	EWGSOP	153(50%)	Sarcopenia in community-dwelling older adults is associated with various factors including age, marital status, disability, behavioral habits, and certain diseases.
Dodds 2017	Community	UK	Cohort study	719	282	437	85.5±0.4	⩾85	EWGSOP	149(20.72%)	Old population revealed a significant prevalence of sarcopenia, with low BMI as a notable risk factor.
Dodds 2020	Community	UK	Cohort study	1686	824	826	-	⩾60	EWGSOP2	328(19.45%)	Probable sarcopenia is prevalent in early old age. SARC-F tool, polypharmacy, osteoarthritis, and inactivity can help identify at-risk individuals.
Thaliany 2015	Community	Brazil	cross-sectional study	-	-	173	74.8 ± 9.9	⩾60	EWGSOP	31（17.8%）	Sarcopenia prevalence is notable in elderly women in a disadvantaged community, particularly in those over 80 years with recent hospitalization.

Erkoyun 2020	Comm unity	Turkey	cross-sectional study	254	114	140	-	⩾65	EWGSOP	153(64.8%)	Sarcopenia risk is prevalent in the elderly population of Balcova district, Turkey, with age and sedentary lifestyle as significant factors.
Gao 2015	Comm unity	China	cross-sectional study	612	254	358	70.6 ± 6.7	⩾60	AWGS	60(9.8%)	Sarcopenia is more prevalent in rural than urban elderly in western China, with age, malnutrition, and rural residence as risk factors.
Hai 2017	Comm unity	China	cross-sectional study	834	415	419	68.5±6.44	⩾60	AWGS	88(10.6%)	Sarcopenia in Chinese older adults is associated with nut consumption frequency and family function, highlighting potential preventive strategies.
He 2022	Comm unity	China	cross-sectional study	1407	581	826	74.82± 6.68	⩾60	AWGS	275(19.55 %)	Sarcopenia is prevalent in Chongming's older population, particularly among males, emphasizing the need for early intervention and prevention strategies.
Hsu 2014	Comm unity	China	cross-sectional study	353	-	-	82.7 ± 5.3	⩾60	EWGSOP	109(30.9%)	Sarcopenia is linked to cognitive impairment and depressive symptoms in older men in a veterans retirement community in Taiwan.
Hu 2017	Comm unity	China	cross-sectional study	607	-	-	70.6±6.6	⩾60	AWGS	112(18.5)	This study suggests a U-shaped relationship between sleep duration and sarcopenia in Chinese older adults, particularly in women.
Ishii 2014	Comm unity	Japan	Cohort study	1971	635	1336	74.65±5.3	⩾65	EWGSOP	359(18.21)	A simple screening test identifies sarcopenia risk accurately in older adults.
Khongsri 2016	Comm unity	Thailand	cross-sectional study	243	62	181	69.7 ± 6.9	⩾60	EWGSOP	74(30.5%)	Sarcopenia prevalence in Thai elders is 30%, higher in older age.
Kim 2015	Comm unity	Japan	Cohort study	-	-	538	78.45± 2.28	⩾75	EWGSOP	213(39.60 %)	Sarcopenia incidence in elderly women: age, BMI, calf circumference, and TUG are predictors.
Kitamura 2021	Comm unity	Japan	Cohort study	1851	916	935	74.15± 5.98	⩾65	AWGS	261(14.10 %)	Japanese sarcopenia prevalence, risks, and outcomes; interaction with muscle components.
Kuo 2019	Comm unity	China	cross-sectional study	731	345	386	74.9±5.35	⩾65	AWGS	50(6.83%)	Taiwanese study on sarcopenia, gender differences, nutrition, and vitamins.
Kurose 2020	Comm unity	Japan	Cohort study	552	173	379	74.6 ± 6.7	⩾60	EWGSOP	123(22.3%)	This study in Japan found sarcopenia in 22.3% of older adults, with age, obesity, hypertension, care certification, fewer daily conversations, and malnutrition as predictors.
Landi 2013	Comm unity	Italy	Cohort study	354	117	237	85.8 ± 4.9	⩾60	EWGSOP	103(29.1%)	In elderly individuals aged 80 or older, anorexia was found to be significantly associated with a higher risk of sarcopenia, even after adjusting for potential confounders.
Lee 2022	Comm unity	korea	Cohort study	1353	631	722	75.75± 3.85	⩾70	EWGSOP	193(14.2%)	In older males, long sleep duration (>8 hours per night) was independently associated with incident sarcopenia, particularly low muscle mass and low muscle strength.

Lu 2022	Community	China	cross-sectional study	1407	-	-	71.9±5.59	⩾65	AWGS	277(19.69)	High prevalence of sarcopenia without obesity (S) and sarcopenic obesity (SO) was observed in older individuals. Risk factors for S included lower BMI, hip circumference, farming, higher HDL-C levels, and shorter sleep duration. SO was associated with aging, male gender, higher BMI, monocyte levels, and longer sleep duration.
Meng 2015	Community	China	cross-sectional study	771	412	359	73.9 ± 6.2	⩾65	EWGSOP	44(5.7%)	This study in Taichung, Taiwan, using European criteria, found varying sarcopenia prevalence and risk factors, highlighting the importance of diagnostic methods.
Mo 2022	Community	China	cross-sectional study	1050	347	703	70.3 ±7.5	⩾60	AWGS	263(25.0%)	A nomogram predicting sarcopenia risk in community-dwelling older adults was developed and validated, aiding early intervention and management.
Momoki 2017	Community	Japan	cross-sectional study	-	-	186	77.7 ± 6.8	⩾65	AWGS	39(20.97%)	Elderly Japanese women found that age, low BMI, locomotive syndrome, and living arrangements were associated with sarcopenia.
Moreira 2019	Community	Brazil	cross-sectional study	745	221	524	76.6±6.9	⩾65	EWGSOP	145(27.67%)	Sarcopenia prevalence varies with cut-off values for muscle mass, handgrip strength, and gait speed, affecting estimates. Advanced age, race, single marital status, low income, and comorbidities were linked to sarcopenia.
Nakamura 2021	Community	Japan	cohort study	2968	-	-	74.2±6.5	⩾65	AWGS	416（14%）	Sarcopenia prevalence in older Japanese adults is around 7%, associated with higher mortality risk, highlighting the need for public health strategies.
Nasimi 2019	Community	Iran	cross-sectional study	501	254	247	70.3±4.60	⩾60	AWGS	104(20.8%)	Sarcopenia prevalence in older adults is 20.8%, associated with factors like age, BMI, nutritional status, and body fat.
Pereira 2022	Community	Brazil	cross-sectional study	132	52	80	70.0 ± 6.3	⩾60	EWGSOP	83(62.7%)	Sarcopenia prevalence and associated factors varied with diagnostic criteria; low SMI doubled mortality risk in older adults.
Perez-Sousa 2021	Community	Colombia	cross-sectional study	5237	2172	3065	70.4±7.8	⩾60	EWGSOP	2435(46.5%)	In a sample of Colombian older adults, probable sarcopenia (defined as weak grip strength) was prevalent, associated with physical inactivity, diabetes, and arthritis, highlighting the need for prevention and intervention.
Samper-Ternent 2017	Community	Colombia	cross-sectional study	1442	562	880	70.7 ±7.7	⩾60	EWGSOP	166(11.5%)	9.4% frailty and 11.5% sarcopenia prevalence among older adults. Both conditions correlated with older age and female gender. Frailty related to depression, while sarcopenia related to smoking. Maintaining independence reduced frailty.
Shibuki 2023	Community	Japan	cross-sectional study	2069	902	1167	71.5±4.05	⩾65	AWGS	209(10.10%)	Long sleep duration is associated with sarcopenia, particularly low muscle strength and physical performance, in older adults in Japan. Poor sleep quality was linked to sarcopenia in normal sleepers but not in long sleepers.
Simsek 2019	Community	Turkey	cross-sectional study	909	327	582	72.8±6.2	⩾65	EWGSOP	47（5.2%）	The prevalence of sarcopenia in individuals aged 65 years and older in the Bornova district of Izmir, Turkey, was 5.2%. Risk factors

											included increasing age, physical inactivity, low body mass index, and malnutrition.
Souza 2019	Community	Brazil	cross-sectional study	1078	-	-	-	⩾60	EWGSOP	101（9.4%）	A study in the Amazon region found a significant association between sarcopenia and diabetes mellitus in older adults.
Souza L 2022	Community	Brazil	cross-sectional study	308	146	162	71.27±7.63	⩾60	EWGSOP	91(29.55%)	Simple sociodemographic and anthropometric factors can effectively screen for sarcopenia in older adults.
Sri-On 2022	Community	Thailand	cross-sectional study	892	278	614	-	⩾60	AWGS	198(22.2%)	About one-third of urban Thai older adults have sarcopenia or severe sarcopenia, with age, low BMI, and poor nutrition as key risk factors.
Su 2019	Community	Japan	cross-sectional study	310	89	221	76.0 ±5.8	⩾60	EWGSOP	45(14.5%)	Sarcopenia prevalence in older adults from snow-covered cities is low. Diabetes and multiple medications are risk factors, while exercise and nutrition are crucial for prevention.
Tanaka 2022	Community	Japan	cohort study	1483	752	731	72.6±5.4	⩾65	AWGS	178(12%)	Social engagement can reduce the risk of new-onset sarcopenia in older adults by influencing factors like physical activity, oral function, psychological and nutritional status.
Tsekoura 2021	Community	Greece	cross-sectional study	402	110	292	71.51±7.63	⩾60	EWGSOP	102（25.37）	The prevalence of probable sarcopenia in older people in Western Greece is 25.4%, associated with age, gender, BMI, muscle mass, calf circumference, and comorbidities.
Tseng 2020	Community	China	cross-sectional study	1025	310	715	73.7±7.0	⩾60	EWGSOP	179(17.5%)	A new prediction model, TRSS, incorporating seven factors, provides an effective community-based screening tool for sarcopenia.
Tsutsumimoto 2020	Community	Japan	cross-sectional study	9496	5033	4463	74.1 ±5.4	⩾60	AWGS	927(9.8%)	Anorexia of aging is independently associated with sarcopenia in elderly Japanese individuals, highlighting the need for further investigation.
Volpato 2014	Community	Italy	cross-sectional study	730	349	381	76.3±4.96	⩾65	EWGSOP	55(7.53%)	Sarcopenia is associated with depression, cognitive impairment, and low physical activity in community-dwelling older adults.
Wang 2016	Community	China	cross-sectional study	1090	520	570	69±7.17	⩾60	AWGS	237(21.74%)	Type 2 diabetes is associated with a higher risk of sarcopenia and pre-sarcopenia in Chinese elderly individuals.
Wu 2014	Community	China	cross-sectional study	549	285	264	76.0 ±6.2	⩾65	EWGSOP	101(18.40)	Approximately one-fifth of rural Taiwanese older adults face the threat of sarcopenia, with factors including age, sex, BMI, and more.
Wu 2021	Community	China	cohort study	6172	3102	3070	68.62±6.26	⩾60	AWGS	2376(38.5%)	factors including age, rural residence, falls, chronic diseases, and inflammation associated with possible sarcopenia.

Wu 2022	Community	China	cross-sectional study	275	69	206	71.9±6.31	⩾60	AWGS	189(68.7%)	A high prevalence of possible sarcopenia and physical inactivity in rural community daycare stations, with poor lower-limb muscle function being a common factor associated with both conditions.
Xing 2022	Community	China	cross-sectional study	158	62	96	68.8±6.5	⩾60	AWGS	32(20.25%)	a high prevalence of sarcopenia among elderly individuals with hypertension, with factors such as advanced age, diabetes, osteoporosis, drinking, and body mass index being associated with sarcopenia in this population.
Yu 2014	Community	China	cross-sectional study	4000	2000	2000	72.5 ± 5.2	⩾65	EWGSOP	217(5.4%)	Sarcopenia incidence rises with age but can be reversible. Factors include age, gender, chronic conditions, activity level, and body mass index.
Yuenyongchaiwat 2020	Community	Thailand	cross-sectional study	330	79	251	66.85± 5.54	⩾60	AWGS	251(76.06 %)	Sarcopenia is a major health issue with multiple consequences, including high healthcare costs, and requires effective prevention and treatment.

Figure S1 Risk of bias graph of the included cross-sectional studies

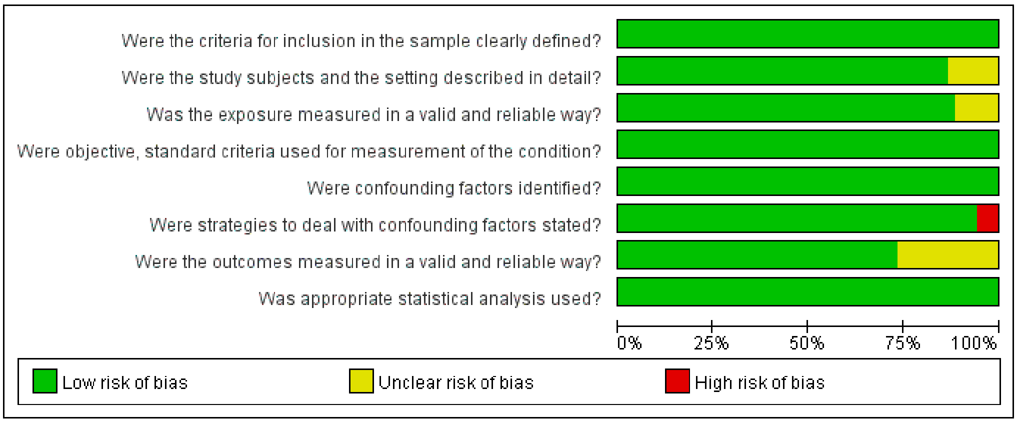

Figure S2 Risk of bias summary of the included cross-sectional studies

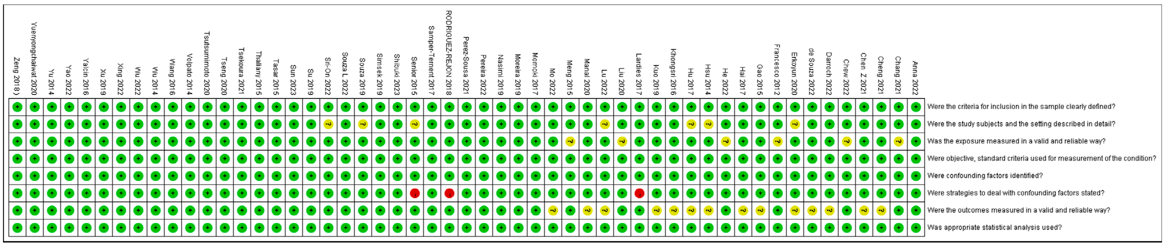

Figure S3 Risk of bias graph of the included cohort studies

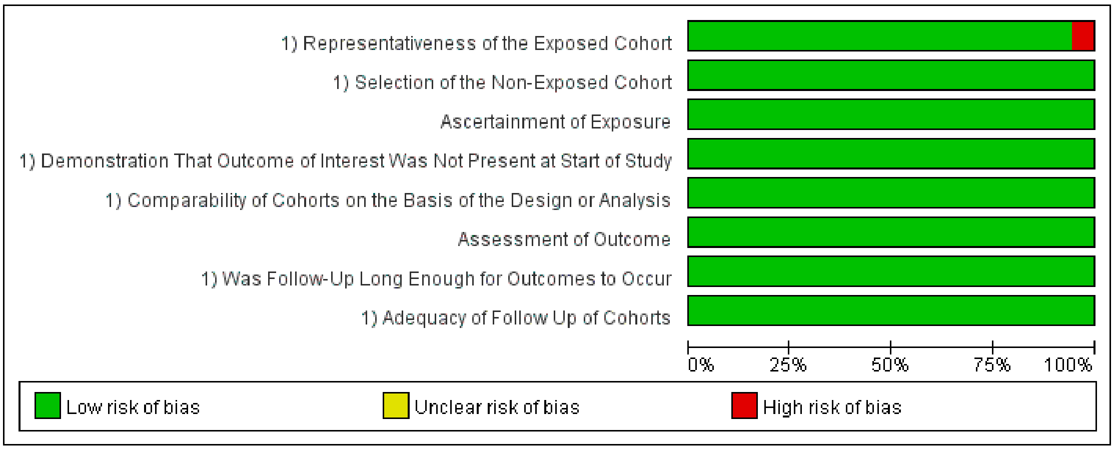

Figure S4 Risk of bias summary of the included cohort studies

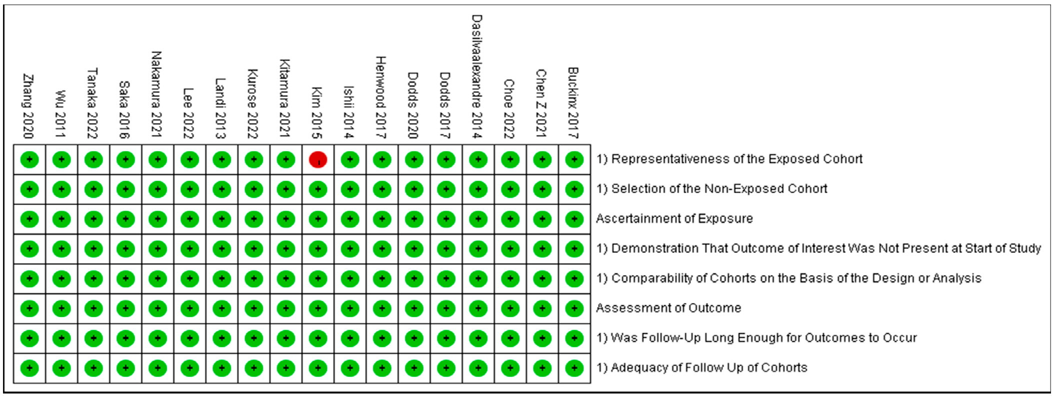

Figure S5 Forest plot of the association between male and sarcopenia in NH.

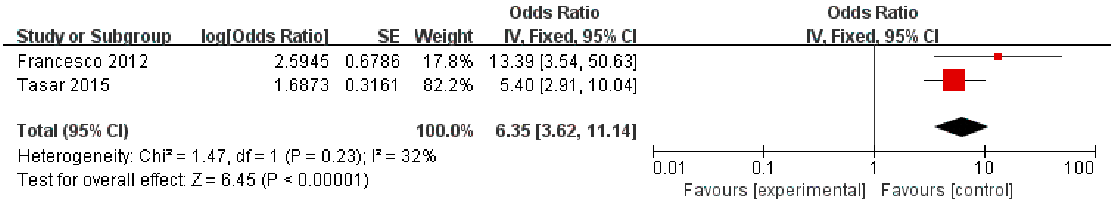

Figure S6 Forest plot of the association between BMI and sarcopenia in NH

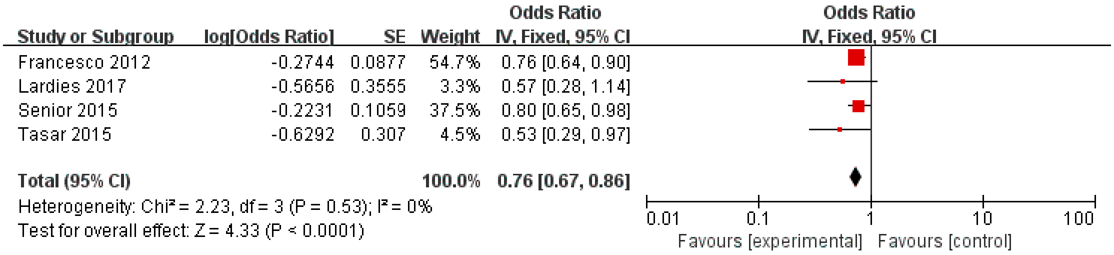

Figure S7 Forest plot of the association between malnutrition and sarcopenia in NH

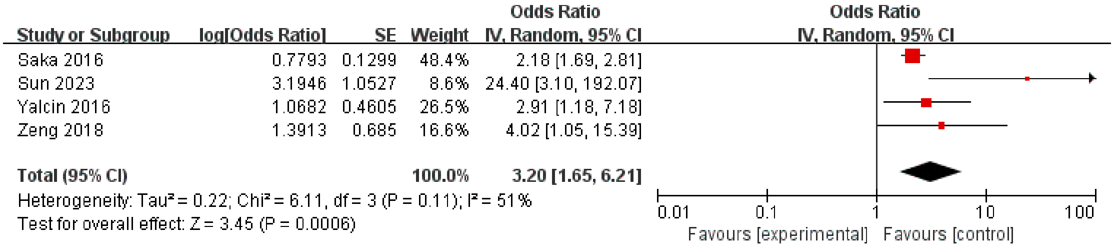

Figure S8 Forest plot of the association between osteoarthritis and sarcopenia in NH

Study or Subgroup	log[Odds Ratio]	SE	Weight	Odds Ratio IV, Random, 95% CI
Francesco 2012	1.9796	0.6513	40.7%	7.24 [2.02, 25.95]
Sun 2023	0.5878	0.2513	59.3%	1.80 [1.10, 2.95]
**Total (95% CI)**			**100.0%**	**3.17 [0.83, 12.11]**

Heterogeneity: Tau² = 0.72; Chi² = 3.97, df = 1 (P = 0.05); I² = 75%
Test for overall effect: Z = 1.69 (P = 0.09)

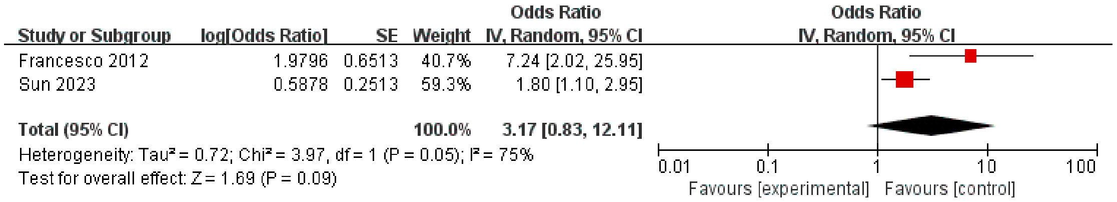

Figure S9 Forest plot of the association between age and sarcopenia in community settings

Study or Subgroup	log[Odds Ratio]	SE	Weight	Odds Ratio IV, Random, 95% CI
Cheng I 2021	0.5188	0.1846	0.9%	1.68 [1.17, 2.41]
Chew 2022	0.0392	0.02	5.5%	1.04 [1.00, 1.08]
Choe 2022	0.157	0.0315	5.0%	1.17 [1.10, 1.24]
Dasilvaalexander 2014	0.6931	0.3004	0.4%	2.00 [1.11, 3.60]
Dutra 2015	0.0296	0.6467	0.1%	1.03 [0.29, 3.66]
Erkoyun 2020	0.2624	0.0852	2.7%	1.30 [1.10, 1.54]
Gao 2015	0.1989	0.0301	5.1%	1.22 [1.15, 1.29]
He 2022	0.8459	0.2747	0.5%	2.33 [1.36, 3.99]
Ishii 2014	0.0677	0.0244	5.3%	1.07 [1.02, 1.12]
Khongsri 2016	2.7389	0.5835	0.1%	15.47 [4.93, 48.55]
Kitamura 2021	0.1823	0.0444	4.4%	1.20 [1.10, 1.31]
Kuo 2019	0.1089	0.0248	5.3%	1.12 [1.06, 1.17]
Kurose 2020	0.7514	0.2904	0.4%	2.12 [1.20, 3.75]
Meng 2015	0.8502	0.3038	0.4%	2.34 [1.29, 4.24]
Mo 2022	1.2134	0.2224	0.7%	3.36 [2.18, 5.20]
Momoki 2017	1.1086	0.6485	0.1%	3.03 [0.85, 10.80]
Moreira 2019	3.6163	0.5626	0.1%	37.20 [12.35, 112.05]
Nakamura 2021	0.1133	0.0186	5.5%	1.12 [1.08, 1.16]
Nasimi 2019	0.0953	0.0385	4.7%	1.10 [1.02, 1.19]
Samper-Ternent 2017	0.0392	0.0099	5.7%	1.04 [1.02, 1.06]
Simsek 2019	0.0825	0.03	5.1%	1.09 [1.02, 1.15]
Souza 2019	0.1124	0.0195	5.5%	1.12 [1.08, 1.16]
Souza L 2022	-0.2877	0.05	4.2%	0.75 [0.68, 0.83]
Sri-On 2022	0.8755	0.185	0.9%	2.40 [1.67, 3.45]
Tsekoura 2021	0.3365	1.4603	0.0%	1.40 [0.08, 24.50]
Tseng 2020	1.2355	0.1923	0.9%	3.44 [2.36, 5.01]
Wu 2011	0.0862	0.0047	5.8%	1.09 [1.08, 1.10]
Wu 2014	0.1071	0.0395	4.7%	1.11 [1.03, 1.20]
Wu 2022	0.0862	0.024	5.3%	1.09 [1.04, 1.14]
Xing 2022	1.0986	0.4804	0.2%	3.00 [1.17, 7.69]
Xu 2020	0.131	0.0371	4.8%	1.14 [1.06, 1.23]
Yu 2014	-0.1054	0.0352	4.9%	0.90 [0.84, 0.96]
Yuenyongchaiwat 2020	1.5412	0.3354	0.3%	4.67 [2.42, 9.01]
Zhang 2020	0.1017	0.0348	4.9%	1.11 [1.03, 1.19]
**Total (95% CI)**			**100.0%**	**1.15 [1.10, 1.19]**

Heterogeneity: Tau² = 0.01; Chi² = 342.35, df = 33 (P < 0.00001); I² = 90%
Test for overall effect: Z = 7.02 (P < 0.00001)

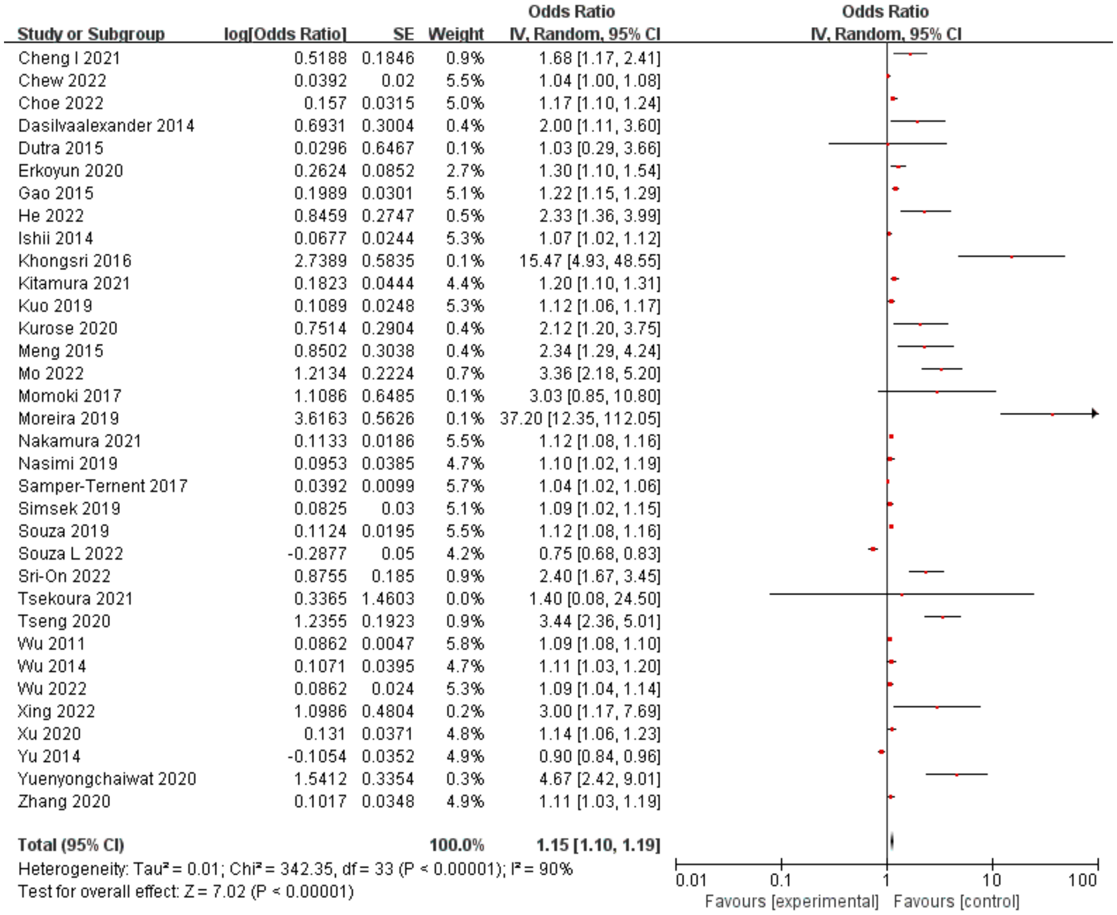

Figure S10 Forest plot of the association between male and sarcopenia in community settings

Study or Subgroup	log[Odds Ratio]	SE	Weight	Odds Ratio IV, Random, 95% CI
Badrasawi 2020	0.6471	0.279	16.5%	1.91 [1.11, 3.30]
Chang 2021	0.6471	0.4869	12.1%	1.91 [0.74, 4.96]
Edith 2005	1.3635	0.6333	9.5%	3.91 [1.13, 13.53]
He 2022	2.2138	0.3145	15.7%	9.15 [4.94, 16.95]
Kuo 2019	0.8887	0.3243	15.5%	2.43 [1.29, 4.59]
Nasimi 2019	1.141	0.4765	12.3%	3.13 [1.23, 7.96]
Samper-Ternent 2017	0.4121	0.1854	18.3%	1.51 [1.05, 2.17]
**Total (95% CI)**			**100.0%**	**2.77 [1.63, 4.69]**

Heterogeneity: Tau² = 0.36; Chi² = 26.13, df = 6 (P = 0.0002); I² = 77%
Test for overall effect: Z = 3.77 (P = 0.0002)

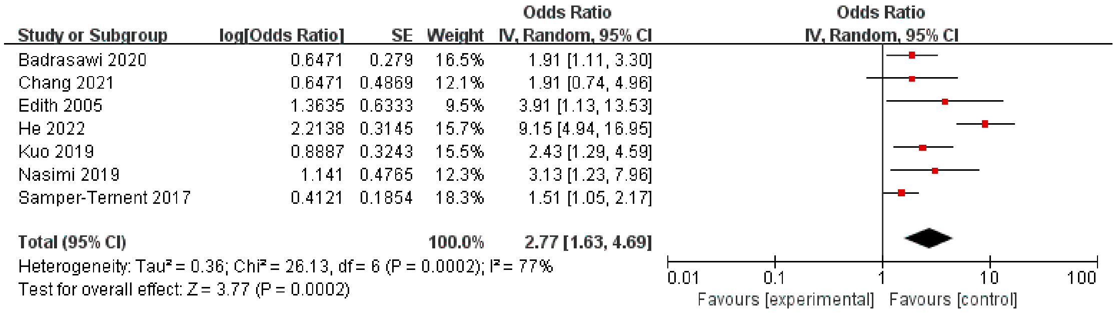

Figure S11 Forest plot of the association between female and sarcopenia in community settings

Study or Subgroup	log[Odds Ratio]	SE	Weight	Odds Ratio IV, Random, 95% CI
Chew 2022	-1.8971	0.3889	33.2%	0.15 [0.07, 0.32]
Meng 2015	0.7275	0.2955	34.2%	2.07 [1.16, 3.69]
Souza 2019	-1.0413	0.4299	32.7%	0.35 [0.15, 0.82]
**Total (95% CI)**			**100.0%**	**0.49 [0.09, 2.55]**

Heterogeneity: Tau² = 2.00; Chi² = 31.59, df = 2 (P < 0.00001); I² = 94%
Test for overall effect: Z = 0.85 (P = 0.39)

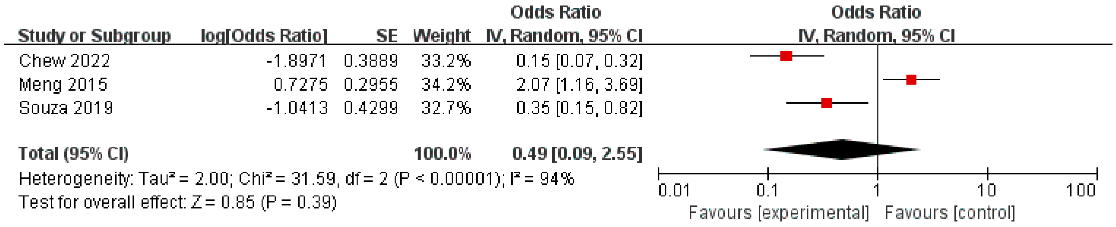

Figure S12 Forest plot of the association between BMI and sarcopenia in community settings

Study or Subgroup	log[Odds Ratio]	SE	Weight	Odds Ratio IV, Random, 95% CI
Chang 2021	-1.5141	0.456	2.9%	0.22 [0.09, 0.54]
Cheng I 2021	-1.3093	0.2999	4.2%	0.27 [0.15, 0.49]
Darroch 2022	0.3365	0.123	5.9%	1.40 [1.10, 1.78]
Dodds 2017	0.2546	0.0327	6.3%	1.29 [1.21, 1.38]
He 2022	-0.2877	0.073	6.2%	0.75 [0.65, 0.87]
Khongsri 2016	2.5526	0.6145	2.0%	12.84 [3.85, 42.82]
Kim 2015	0.8372	0.3384	3.8%	2.31 [1.19, 4.48]
Kuo 2019	-0.3857	0.0596	6.2%	0.68 [0.61, 0.76]
Lu 2022	-1.9951	0.4713	2.8%	0.14 [0.05, 0.34]
Mo 2022	0.8965	0.4138	3.2%	2.45 [1.09, 5.52]
Momoki 2017	1.6845	0.7794	1.4%	5.39 [1.17, 24.83]
Nasimi 2019	2.3646	0.8042	1.3%	10.64 [2.20, 51.46]
Simsek 2019	0.9104	0.456	2.9%	2.49 [1.02, 6.07]
Souza 2019	-0.3769	0.178	5.4%	0.69 [0.48, 0.97]
Souza L 2022	-0.1278	0.0282	6.3%	0.88 [0.83, 0.93]
Sri-On 2022	2.1736	0.3485	3.7%	8.79 [4.44, 17.40]
Su 2019	-0.2877	0.0971	6.0%	0.75 [0.62, 0.91]
Tsekoura 2021	-4.6052	0.3537	3.7%	0.01 [0.00, 0.02]
Tseng 2020	0.6152	0.3088	4.1%	1.85 [1.01, 3.39]
Wu 2014	-0.3481	0.1453	5.7%	0.71 [0.53, 0.94]
Xing 2022	-0.3011	0.1156	5.9%	0.74 [0.59, 0.93]
Xu 2020	-0.2231	0.0467	6.3%	0.80 [0.73, 0.88]
Zhang 2020	-1.5233	0.3584	3.7%	0.22 [0.11, 0.44]
**Total (95% CI)**			**100.0%**	**0.83 [0.68, 1.02]**

Heterogeneity: Tau² = 0.17; Chi² = 497.57, df = 22 (P < 0.00001); I² = 96%
Test for overall effect: Z = 1.74 (P = 0.08)

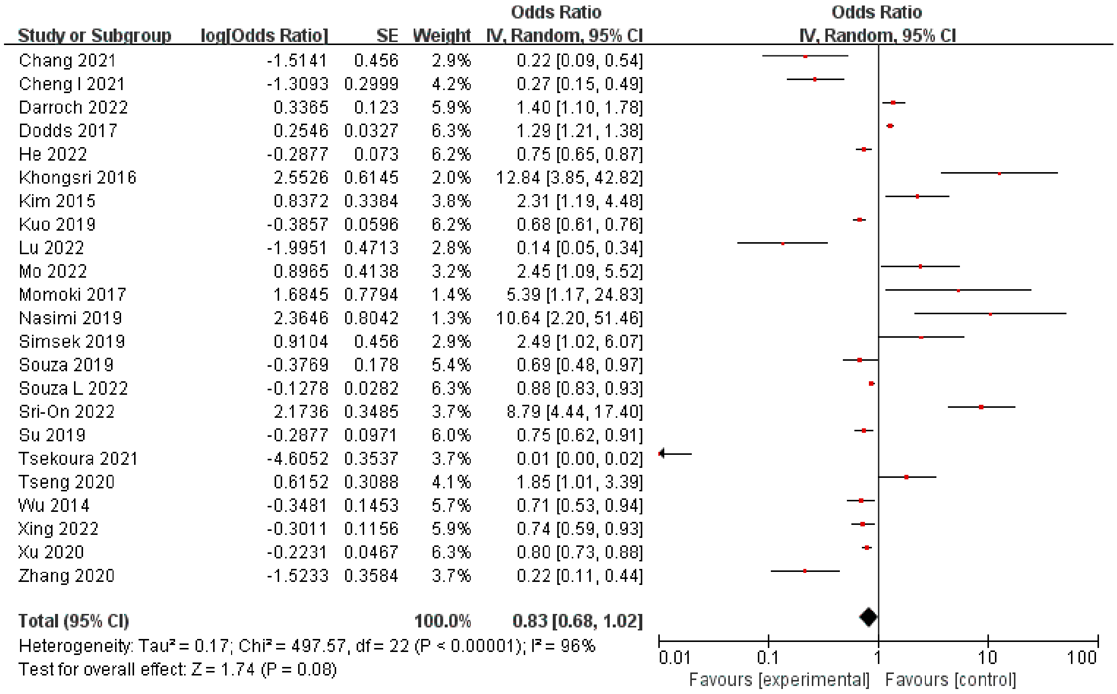

Figure S13 Forest plot of the association between malnutrition and sarcopenia in community settings

Study or Subgroup	log[Odds Ratio]	SE	Weight	Odds Ratio IV, Random, 95% CI
Chang 2021	1.7317	0.5402	9.3%	5.65 [1.96, 16.29]
Cheng I 2021	0.8109	0.325	11.5%	2.25 [1.19, 4.25]
Darroch 2022	0.47	0.2398	12.3%	1.60 [1.00, 2.56]
Dasilvaalexander 2014	1.1474	0.2242	12.4%	3.15 [2.03, 4.89]
Gao 2015	1.2613	0.3788	11.0%	3.53 [1.68, 7.42]
Kuo 2019	1.0505	0.4723	10.0%	2.86 [1.13, 7.22]
Kurose 2020	0.8838	0.4309	10.4%	2.42 [1.04, 5.63]
Simsek 2019	2.0802	0.4892	9.8%	8.01 [3.07, 20.88]
Xu 2020	-0.2485	0.1009	13.2%	0.78 [0.64, 0.95]
**Total (95% CI)**			**100.0%**	**2.62 [1.47, 4.67]**

Heterogeneity: Tau² = 0.65; Chi² = 77.80, df = 8 (P < 0.00001); I² = 90%
Test for overall effect: Z = 3.25 (P = 0.001)

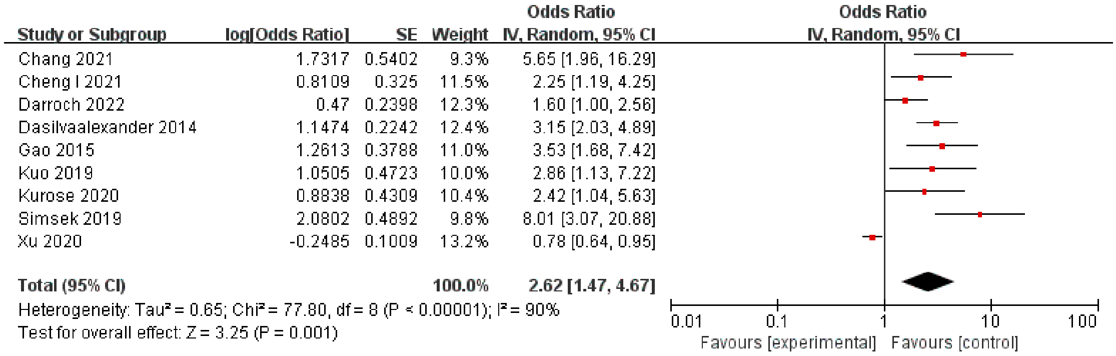

Figure S14 Forest plot of the association between nutrition status and sarcopenia in community settings

Study or Subgroup	log[Odds Ratio]	SE	Weight	Odds Ratio IV, Fixed, 95% CI
Choe 2022	-0.2357	0.0617	53.0%	0.79 [0.70, 0.89]
Hai 2017	-0.323	0.1572	8.2%	0.72 [0.53, 0.99]
Wu 2014	-0.1543	0.0721	38.8%	0.86 [0.74, 0.99]
**Total (95% CI)**			**100.0%**	**0.81 [0.74, 0.88]**

Heterogeneity: Chi² = 1.29, df = 2 (P = 0.53); I² = 0%
Test for overall effect: Z = 4.70 (P < 0.00001)

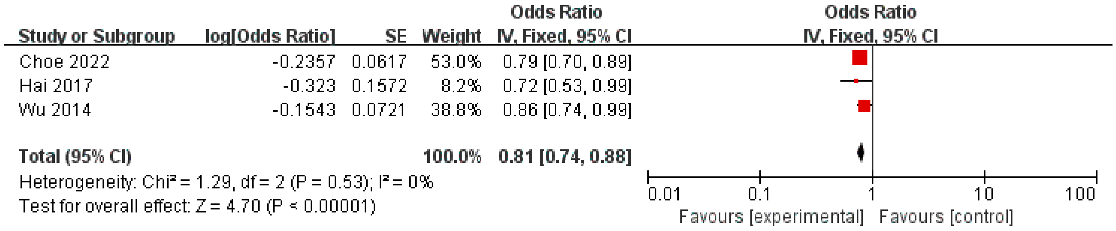

Figure S15 Forest plot of the association between calf circumference and sarcopenia in community settings

Study or Subgroup	log[Odds Ratio]	SE	Weight	Odds Ratio IV, Random, 95% CI
Chang 2021	1.2208	0.4194	12.5%	3.39 [1.49, 7.71]
Chew 2022	-0.2485	0.0552	26.6%	0.78 [0.70, 0.87]
Ishii 2014	-0.478	0.0519	26.6%	0.62 [0.56, 0.69]
Nasimi 2019	-0.6931	0.1139	25.0%	0.50 [0.40, 0.63]
Tsekoura 2021	-3.912	0.5356	9.3%	0.02 [0.01, 0.06]
**Total (95% CI)**			**100.0%**	**0.56 [0.38, 0.83]**

Heterogeneity: Tau² = 0.15; Chi² = 74.31, df = 4 (P < 0.00001); I² = 95%
Test for overall effect: Z = 2.86 (P = 0.004)

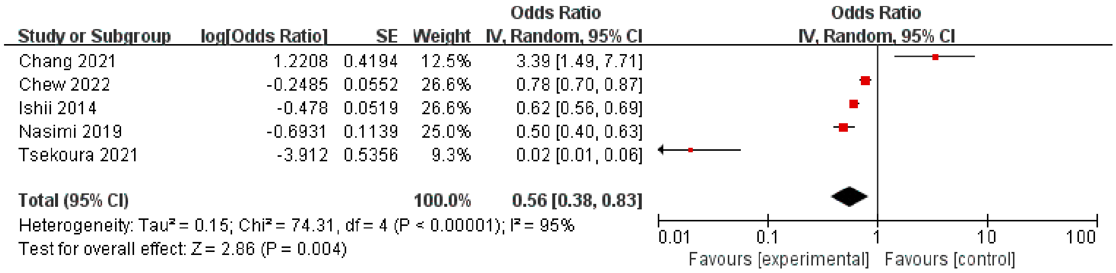

Figure S16 Forest plot of the association between smoking and sarcopenia in community settings

Study or Subgroup	log[Odds Ratio]	SE	Weight	Odds Ratio IV, Fixed, 95% CI
Dasilvaalexander 2014	0.6931	0.3004	27.7%	2.00 [1.11, 3.60]
Kuo 2019	0.7738	0.348	20.6%	2.17 [1.10, 4.29]
Samper-Ternent 2017	0.8671	0.3125	25.6%	2.38 [1.29, 4.39]
Tseng 2020	0.6152	0.3088	26.2%	1.85 [1.01, 3.39]
**Total (95% CI)**			**100.0%**	**2.08 [1.53, 2.84]**

Heterogeneity: Chi² = 0.36, df = 3 (P = 0.95); I² = 0%
Test for overall effect: Z = 4.64 (P < 0.00001)

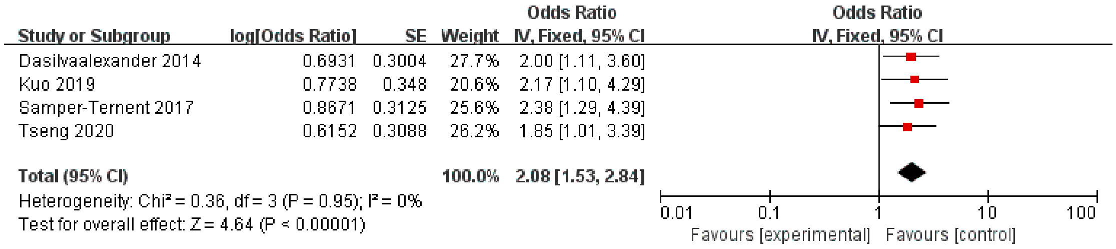

Figure S17 Forest plot of the association between physical inactivity and sarcopenia in communitysettings

Study or Subgroup	log[Odds Ratio]	SE	Weight	Odds Ratio IV, Random, 95% CI
Dodds 2020	0.7419	0.1717	14.1%	2.10 [1.50, 2.94]
Erkoyun 2020	1.1314	0.4434	5.3%	3.10 [1.30, 7.39]
Kitamura 2021	0.3365	0.1717	14.1%	1.40 [1.00, 1.96]
Meng 2015	0.6419	0.2978	8.9%	1.90 [1.06, 3.41]
Mo 2022	0.675	0.2574	10.3%	1.96 [1.19, 3.25]
Perez-Sousa 2021	0.3001	0.0863	18.1%	1.35 [1.14, 1.60]
Simsek 2019	1.1823	0.5759	3.5%	3.26 [1.06, 10.08]
Wu 2022	1.2499	0.3464	7.4%	3.49 [1.77, 6.88]
Yao 2022	1.0221	0.2672	10.0%	2.78 [1.65, 4.69]
Yuenyongchaiwat 2020	0.9203	0.3202	8.2%	2.51 [1.34, 4.70]
**Total (95% CI)**			**100.0%**	**2.03 [1.60, 2.56]**

Heterogeneity: Tau² = 0.07; Chi² = 22.57, df = 9 (P = 0.007); I² = 60%
Test for overall effect: Z = 5.91 (P < 0.00001)

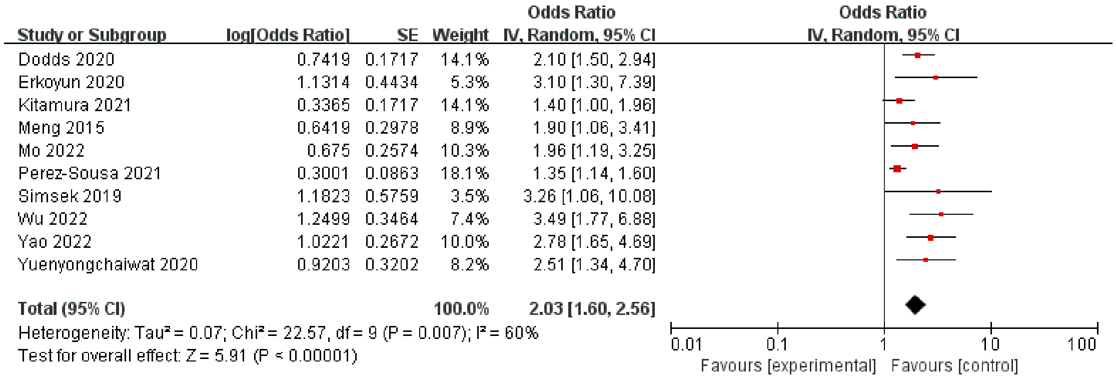

Figure S18 Forest plot of the association between cognitive impairment and sarcopenia in community settings

Study or Subgroup	log[Odds Ratio]	SE	Weight	Odds Ratio IV, Random, 95% CI
Chang 2021	0.8372	0.4171	8.9%	2.31 [1.02, 5.23]
Chen X 2021	1.7599	0.6415	4.9%	5.81 [1.65, 20.43]
Dasilvaalexander 2014	0.9858	0.3974	9.5%	2.68 [1.23, 5.84]
Dodds 2017	0.4947	0.2179	16.0%	1.64 [1.07, 2.51]
Hsu 2014	1.1086	0.3163	12.1%	3.03 [1.63, 5.63]
Kitamura 2021	0.9555	0.2806	13.4%	2.60 [1.50, 4.51]
Souza 2022	0.9123	0.2724	13.7%	2.49 [1.46, 4.25]
Sri-On 2022	0.4187	0.1744	18.0%	1.52 [1.08, 2.14]
Yuenyongchaiwat 2020	-1.4697	0.7786	3.5%	0.23 [0.05, 1.06]
**Total (95% CI)**			**100.0%**	**2.10 [1.54, 2.87]**

Heterogeneity: Tau² = 0.11; Chi² = 17.69, df = 8 (P = 0.02); I² = 55%
Test for overall effect: Z = 4.66 (P < 0.00001)

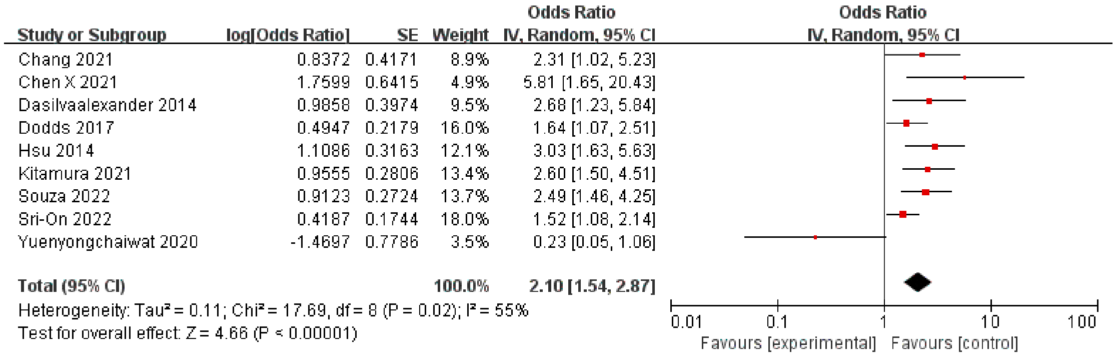

Figure S19 Forest plot of the association between diabetes and sarcopenia in community settings

Study or Subgroup	log[Odds Ratio]	SE	Weight	Odds Ratio IV, Random, 95% CI
Perez-Sousa 2021	0.2776	0.0884	24.5%	1.32 [1.11, 1.57]
Souza 2019	1.1656	0.2994	17.6%	3.21 [1.78, 5.77]
Su 2019	1.2975	0.6323	8.5%	3.66 [1.06, 12.64]
Wang 2016	0.3148	0.1505	22.9%	1.37 [1.02, 1.84]
Xing 2022	1.4929	0.62	8.7%	4.45 [1.32, 15.00]
Yao 2022	1.4514	0.2945	17.8%	4.27 [2.40, 7.60]
**Total (95% CI)**			**100.0%**	**2.33 [1.49, 3.62]**

Heterogeneity: Tau² = 0.20; Chi² = 26.44, df = 5 (P < 0.0001); I² = 81%
Test for overall effect: Z = 3.73 (P = 0.0002)

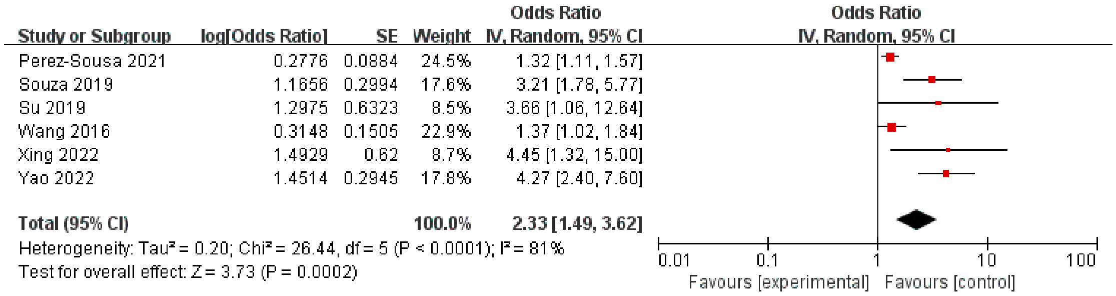

Figure S20 Forest plot of the association between depression and sarcopenia in community settings

Study or Subgroup	log[Odds Ratio]	SE	Weight	Odds Ratio IV, Fixed, 95% CI
Chen Z 2021	0.5241	0.1273	43.6%	1.69 [1.32, 2.17]
He 2022	0.9746	0.3404	6.1%	2.65 [1.36, 5.16]
Hsu 2014	0.8109	0.3987	4.4%	2.25 [1.03, 4.92]
Kitamura 2021	0.6419	0.1558	29.1%	1.90 [1.40, 2.58]
Souza 2022	0.7885	0.2606	10.4%	2.20 [1.32, 3.67]
Yuenyongchaiwat 2020	0.8502	0.3323	6.4%	2.34 [1.22, 4.49]
**Total (95% CI)**			**100.0%**	**1.91 [1.62, 2.25]**

Heterogeneity: Chi² = 2.70, df = 5 (P = 0.75); I² = 0%
Test for overall effect: Z = 7.70 (P < 0.00001)

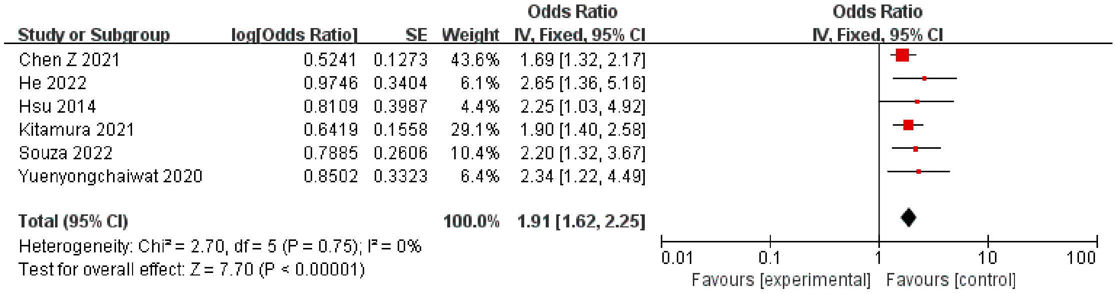

Figure S21 Forest plot of the association between heart diseases and sarcopenia in community settings

Study or Subgroup	log[Odds Ratio]	SE	Weight	Odds Ratio IV, Random, 95% CI
Chen X 2021	2.9204	1.2136	8.5%	18.55 [1.72, 200.14]
Kim 2015	0.6916	0.2996	39.9%	2.00 [1.11, 3.59]
Wu 2011	0.1398	0.0713	51.6%	1.15 [1.00, 1.32]
**Total (95% CI)**			**100.0%**	**1.81 [0.85, 3.86]**

Heterogeneity: Tau² = 0.28; Chi² = 8.33, df = 2 (P = 0.02); I² = 76%
Test for overall effect: Z = 1.54 (P = 0.12)

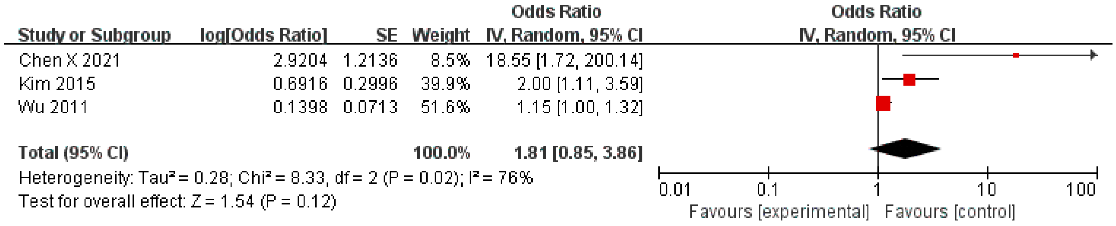

Figure S22 Forest plot of the association between osteoarthritis and sarcopenia in community settings

Study or Subgroup	log[Odds Ratio]	SE	Weight	Odds Ratio IV, Random, 95% CI
Dodds 2020	0.5878	0.166	17.0%	1.80 [1.30, 2.49]
Kuo 2019	0.7031	0.3023	8.2%	2.02 [1.12, 3.65]
Perez-Sousa 2021	0.3646	0.0722	27.3%	1.44 [1.25, 1.66]
Wu 2011	0.1906	0.0627	28.3%	1.21 [1.07, 1.37]
Xing 2022	0.9243	0.4092	5.0%	2.52 [1.13, 5.62]
Yao 2022	0.7304	0.197	14.3%	2.08 [1.41, 3.05]
**Total (95% CI)**			**100.0%**	**1.59 [1.30, 1.93]**

Heterogeneity: Tau² = 0.03; Chi² = 15.34, df = 5 (P = 0.009); I² = 67%
Test for overall effect: Z = 4.61 (P < 0.00001)

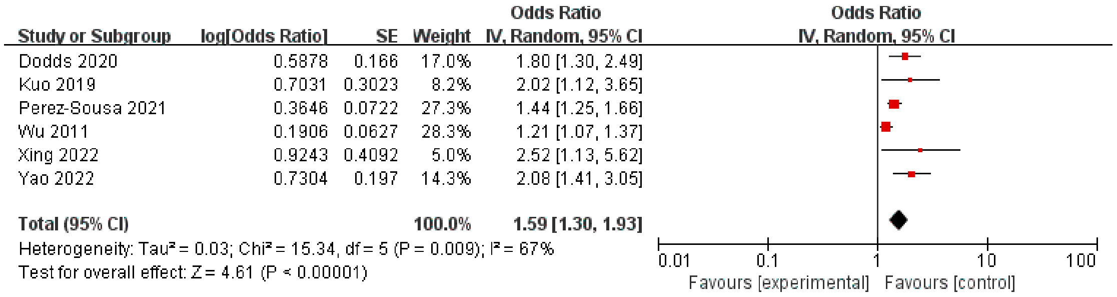

Figure S23 Forest plot of the association between male and sarcopenia in nursing home and community settings

Study or Subgroup	log[Odds Ratio]	SE	Weight	Odds Ratio IV, Random, 95% CI
Badrasawi 2020	0.6471	0.279	13.0%	1.91 [1.11, 3.30]
Chang 2021	0.6471	0.4869	9.9%	1.91 [0.74, 4.96]
Edith 2005	1.3635	0.6333	8.0%	3.91 [1.13, 13.53]
Francesco 2012	2.5945	0.6786	7.4%	13.39 [3.54, 50.63]
He 2022	2.2138	0.3145	12.5%	9.15 [4.94, 16.95]
Kuo 2019	0.8887	0.3243	12.4%	2.43 [1.29, 4.59]
Nasimi 2019	1.141	0.4765	10.1%	3.13 [1.23, 7.96]
Samper-Ternent 2017	0.4121	0.1854	14.2%	1.51 [1.05, 2.17]
Tasar 2015	1.6873	0.3161	12.5%	5.40 [2.91, 10.04]
**Total (95% CI)**			**100.0%**	**3.39 [2.05, 5.62]**

Heterogeneity: Tau² = 0.43; Chi² = 37.86, df = 8 (P < 0.00001); I² = 79%
Test for overall effect: Z = 4.73 (P < 0.00001)

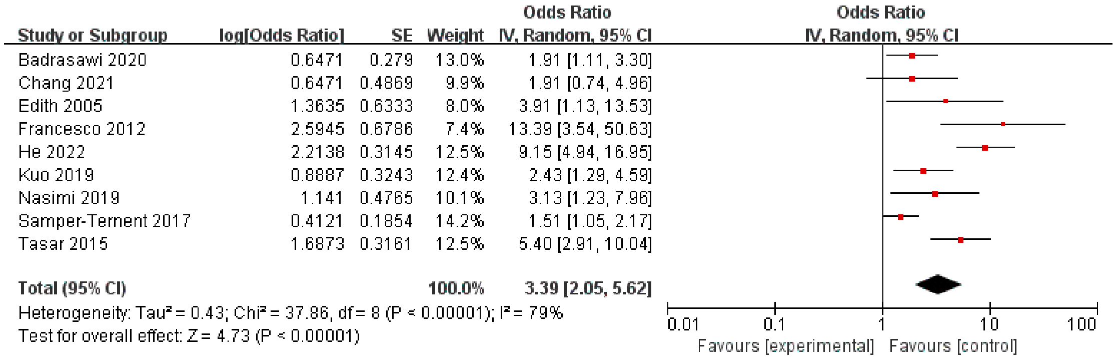

Figure S24 Forest plot of the association between BMI and sarcopenia in nursing home and community settings

Study or Subgroup	log[Odds Ratio]	SE	Weight	Odds Ratio IV, Random, 95% CI
Chang 2021	-1.5141	0.456	2.4%	0.22 [0.09, 0.54]
Cheng I 2021	-1.3093	0.2999	3.5%	0.27 [0.15, 0.49]
Darroch 2022	0.3365	0.123	4.9%	1.40 [1.10, 1.78]
Dodds 2017	0.2546	0.0327	5.4%	1.29 [1.21, 1.38]
Francesco 2012	-0.2744	0.0877	5.2%	0.76 [0.64, 0.90]
He 2022	-0.2877	0.073	5.2%	0.75 [0.65, 0.87]
Khongsri 2016	2.5526	0.6145	1.6%	12.84 [3.85, 42.82]
Kim 2015	0.8372	0.3384	3.2%	2.31 [1.19, 4.48]
Kuo 2019	-0.3857	0.0596	5.3%	0.68 [0.61, 0.76]
Lardies 2017	-0.5656	0.3555	3.0%	0.57 [0.28, 1.14]
Lu 2022	-1.9951	0.4713	2.3%	0.14 [0.05, 0.34]
Mo 2022	0.8965	0.4138	2.6%	2.45 [1.09, 5.52]
Momoki 2017	1.6845	0.7794	1.1%	5.39 [1.17, 24.83]
Nasimi 2019	2.3646	0.8042	1.1%	10.64 [2.20, 51.46]
Senior 2015	-0.2231	0.1059	5.0%	0.80 [0.65, 0.98]
Simsek 2019	0.9104	0.456	2.4%	2.49 [1.02, 6.07]
Souza 2019	-0.3769	0.178	4.5%	0.69 [0.48, 0.97]
Souza L 2022	-0.1278	0.0282	5.4%	0.88 [0.83, 0.93]
Sri-On 2022	2.1736	0.3485	3.1%	8.79 [4.44, 17.40]
Su 2019	-0.2877	0.0971	5.1%	0.75 [0.62, 0.91]
Tasar 2015	-0.6292	0.307	3.4%	0.53 [0.29, 0.97]
Tsekoura 2021	-4.6052	0.3537	3.0%	0.01 [0.00, 0.02]
Tseng 2020	0.6152	0.3088	3.4%	1.85 [1.01, 3.39]
Wu 2014	-0.3481	0.1453	4.8%	0.71 [0.53, 0.94]
Xing 2022	-0.3011	0.1156	5.0%	0.74 [0.59, 0.93]
Xu 2020	-0.2231	0.0467	5.3%	0.80 [0.73, 0.88]
Zhang 2020	-1.5233	0.3584	3.0%	0.22 [0.11, 0.44]
**Total (95% CI)**			**100.0%**	**0.80 [0.67, 0.96]**

Heterogeneity: Tau² = 0.16; Chi² = 509.03, df = 26 (P < 0.00001); I² = 95%
Test for overall effect: Z = 2.35 (P = 0.02)

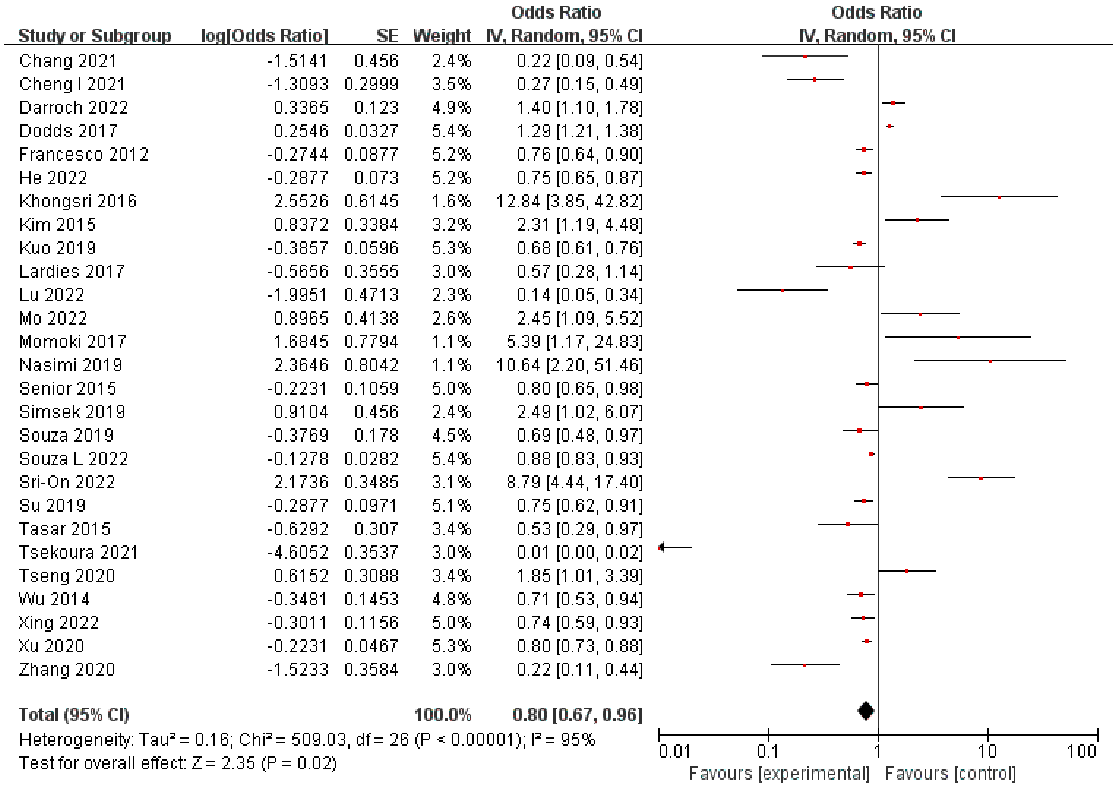

Figure S25 Forest plot of the association between malnutrition and sarcopenia in nursing home and community settings

Study or Subgroup	log[Odds Ratio]	SE	Weight	Odds Ratio IV, Random, 95% CI
Chang 2021	1.7317	0.5402	6.6%	5.65 [1.96, 16.29]
Cheng I 2021	0.8109	0.325	8.6%	2.25 [1.19, 4.25]
Darroch 2022	0.47	0.2398	9.3%	1.60 [1.00, 2.56]
Dasilvaalexander 2014	1.1474	0.2242	9.5%	3.15 [2.03, 4.89]
Gao 2015	1.2613	0.3788	8.1%	3.53 [1.68, 7.42]
Kuo 2019	1.0505	0.4723	7.2%	2.86 [1.13, 7.22]
Kurose 2020	0.8838	0.4309	7.6%	2.42 [1.04, 5.63]
Saka 2016	0.7793	0.1299	10.1%	2.18 [1.69, 2.81]
Simsek 2019	2.0802	0.4892	7.0%	8.01 [3.07, 20.88]
Sun 2023	3.1946	1.0527	3.2%	24.40 [3.10, 192.07]
Xu 2020	-0.2485	0.1009	10.2%	0.78 [0.64, 0.95]
Yalcin 2016	1.0682	0.4605	7.3%	2.91 [1.18, 7.18]
Zeng 2018	1.3913	0.685	5.4%	4.02 [1.05, 15.39]
**Total (95% CI)**			**100.0%**	**2.82 [1.80, 4.40]**

Heterogeneity: Tau² = 0.50; Chi² = 99.69, df = 12 (P < 0.00001); I² = 88%
Test for overall effect: Z = 4.55 (P < 0.00001)

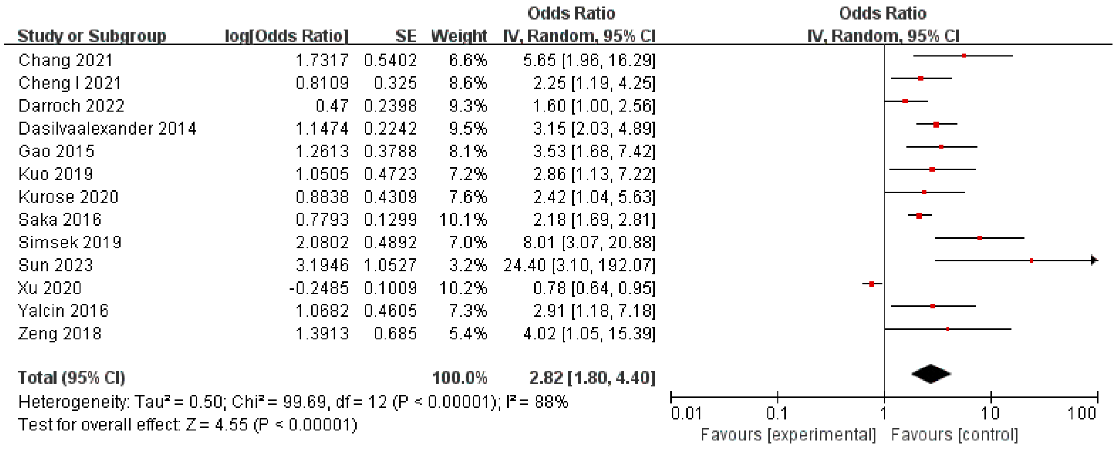

Figure S26 Forest plot of the association between calf circumference and sarcopenia in nursing home and community settings

Study or Subgroup	log[Odds Ratio]	SE	Weight	Odds Ratio IV, Random, 95% CI
Chang 2021	1.2208	0.4194	12.0%	3.39 [1.49, 7.71]
Chew 2022	-0.2485	0.0552	24.6%	0.78 [0.70, 0.87]
Ishii 2014	-0.478	0.0519	24.6%	0.62 [0.56, 0.69]
Nasimi 2019	-0.6931	0.1139	23.2%	0.50 [0.40, 0.63]
Tsekoura 2021	-3.912	0.5356	9.1%	0.02 [0.01, 0.06]
Zeng 2018	1.3913	0.685	6.4%	4.02 [1.05, 15.39]
**Total (95% CI)**			**100.0%**	**0.63 [0.43, 0.94]**

Heterogeneity: Tau² = 0.16; Chi² = 81.18, df = 5 (P < 0.00001); I² = 94%
Test for overall effect: Z = 2.27 (P = 0.02)

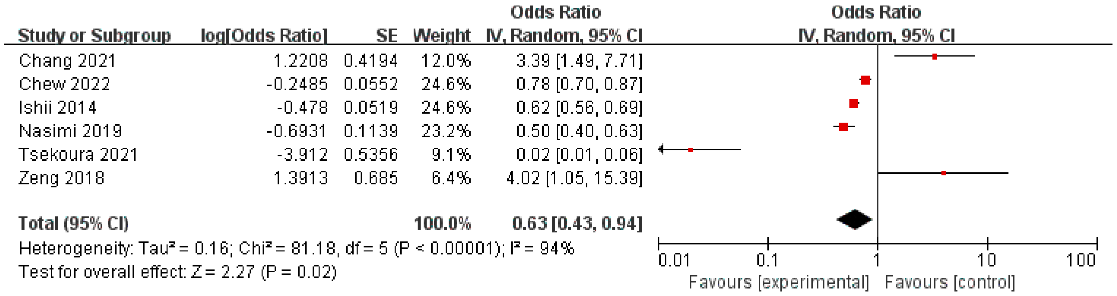

Figure S27 Forest plot of the association between smoking and sarcopenia in nursing home and community settings

Study or Subgroup	log[Odds Ratio]	SE	Weight	Odds Ratio IV, Fixed, 95% CI
Dasilvaalexander 2014	0.6931	0.3004	22.7%	2.00 [1.11, 3.60]
Kuo 2019	0.7738	0.348	16.9%	2.17 [1.10, 4.29]
Samper-Ternent 2017	0.8671	0.3125	21.0%	2.38 [1.29, 4.39]
Tasar 2015	0.675	0.3383	17.9%	1.96 [1.01, 3.81]
Tseng 2020	0.6152	0.3088	21.5%	1.85 [1.01, 3.39]
**Total (95% CI)**			**100.0%**	**2.06 [1.56, 2.73]**

Heterogeneity: Chi² = 0.39, df = 4 (P = 0.98); I² = 0%
Test for overall effect: Z = 5.05 (P < 0.00001)

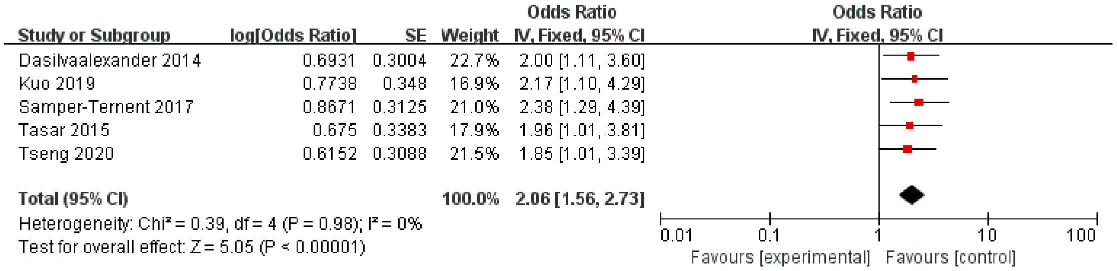

Figure S28 Forest plot of the association between osteoarthritis and sarcopenia in nursing home and community settings

Study or Subgroup	log[Odds Ratio]	SE	Weight	Odds Ratio IV, Random, 95% CI
Dodds 2020	0.5878	0.166	15.4%	1.80 [1.30, 2.49]
Francesco 2012	1.9796	0.6513	2.4%	7.24 [2.02, 25.95]
Kuo 2019	0.7031	0.3023	8.2%	2.02 [1.12, 3.65]
Perez-Sousa 2021	0.3646	0.0722	22.3%	1.44 [1.25, 1.66]
Sun 2023	0.5878	0.2513	10.3%	1.80 [1.10, 2.95]
Wu 2011	0.1906	0.0627	22.9%	1.21 [1.07, 1.37]
Xing 2022	0.9243	0.4092	5.2%	2.52 [1.13, 5.62]
Yao 2022	0.7304	0.197	13.3%	2.08 [1.41, 3.05]
**Total (95% CI)**			**100.0%**	**1.69 [1.38, 2.08]**

Heterogeneity: Tau² = 0.04; Chi² = 22.74, df = 7 (P = 0.002); I² = 69%
Test for overall effect: Z = 4.99 (P < 0.00001)

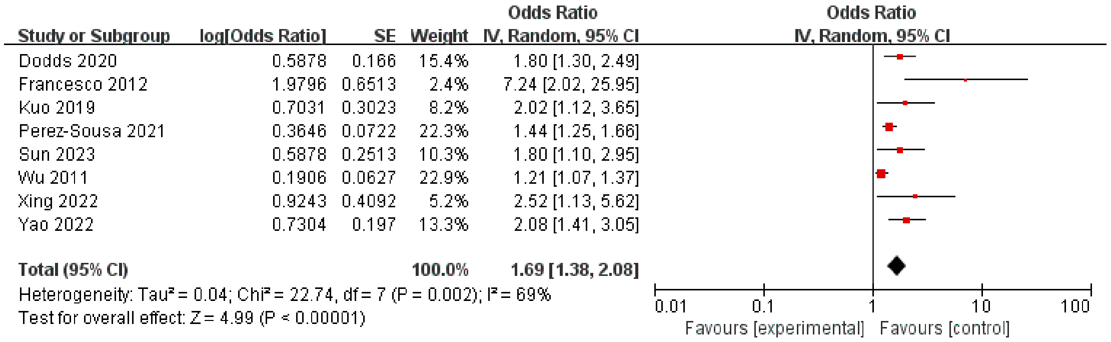

Supplement: Supplementary file 1 [file nutrients-15-04335-s001.zip › nutrients-2593114-supplementary.pdf]
